# CTRP1 prevents high fat diet-induced obesity and improves glucose homeostasis in obese and STZ-induced diabetic mice

**DOI:** 10.1186/s12967-022-03672-5

**Published:** 2022-10-04

**Authors:** Mingzhi Ren, Jianfei Pan, Xueying Yu, Kaile Chang, Xiaopeng Yuan, Chunbo Zhang

**Affiliations:** School of Pharmacy, Nanchang University, Nanchang, 330031 Jiangxi China

**Keywords:** CTRP1, Hydrodynamic gene delivery, Glucose homeostasis, Fatty liver

## Abstract

**Background:**

C1q/tumor necrosis factor-related protein 1 (CTRP1) is an adipokine secreted by adipose tissue, related to chondrocyte proliferation, inflammation, and glucose homeostasis. However, the therapeutic effects on metabolic disorders and the underlying mechanism were unclear. Here, we investigated the functions and mechanisms of CTRP1 in treating obesity and diabetes.

**Methods:**

The plasmid containing human *CTRP1* was delivered to mice by hydrodynamic injection, which sustained expression of *CTRP1* in the liver and high protein level in the blood. High-fat diet (HFD) fed mice and STZ-induced diabetes model were used to study the effects of CTRP1 on obesity, glucose homeostasis, insulin resistance, and hepatic lipid accumulation. The lipid accumulation in liver and adipose tissue, glucose tolerance, insulin sensitivity, food intake, and energy expenditure were detected by H&E staining, Oil-Red O staining, glucose tolerance test, insulin tolerance test, and metabolic cage, respectively. The metabolic-related genes and signal pathways were determined using qPCR and western blotting.

**Results:**

With high blood circulation, CTRP1 prevented obesity, hyperglycemia, insulin resistance, and fatty liver in HFD-fed mice. CTRP1 also improved glucose metabolism and insulin resistance in obese and STZ-induced diabetic mice. The metabolic cage study revealed that CTRP1 reduced food intake and enhanced energy expenditure. The mechanistic study demonstrated that CTRP1 upregulated the protein level of leptin in blood, thermogenic gene expression in brown adipose tissue, and the gene expression responsible for lipolysis and glycolysis in white adipose tissue (WAT). CTRP1 also downregulated the expression of inflammatory genes in WAT. Overexpression of *CTRP1* activated AMPK and PI3K/Akt signaling pathways and inhibited ERK signaling pathway.

**Conclusion:**

These results demonstrate that CTRP1 could improve glucose homeostasis and prevent HFD-induced obesity and fatty liver through upregulating the energy expenditure and reducing food intake, suggesting CTRP1 may serve as a promising target for treating metabolic diseases.

**Supplementary Information:**

The online version contains supplementary material available at 10.1186/s12967-022-03672-5.

## Introduction

C1q/TNF-related proteins (CTRP) are a family of secreted proteins [1]. The protein structures of the CTRP family are similar to that of adiponectin [2]. CTRP1, a 281 amino acids-glycoprotein, is an important adipokine closely related to metabolic and inflammatory pathways [3, 4]. *CTRP1* is expressed in adipose, placenta, and many other tissues [2, 5, 6]. In clinical research, serum CTRP1 level was higher in metabolic syndrome and type II diabetes (T2DM) patients [7–9]. CTRP1 was positively correlated with body mass index (BMI), HbA1c, and blood glucose in metabolic syndrome and T2DM patients [7–9]. High CTRP1 level was also found in patients with nonalcoholic fatty liver disease (NAFLD) and obese people with chronic kidney disease (CKD) [10, 11]. In addition, high CTRP1 expression was detected in Zucker diabetic fatty rats, *ob/ob* mice, and HFD-fed rats [3, 5, 12], suggesting the potential role of CTRP1 in metabolic disorders.

As a paralog of secreted proteins adiponectin, CTRP1 was regarded as a potential target to treat metabolic disease in recent studies. The protein level of CTRP1 was increased in adiponectin knockout (KO) mice to compensate for the function of adiponectin [5]. *Ctrp1* transgenic mice ameliorated insulin resistance (IR) and enhanced fatty acid oxidation and energy consumption [13, 14]. CTRP1 improved the glucose utilization rate in insulin-treated mature adipocytes and stimulated the phosphorylation of insulin receptor substrate (IRS)-1 Ser1101 [15]. *Ctrp1* KO mice showed insulin resistance and higher *G6pc* and *Pck1* expression on a low-fat diet (LFD) [16]. LFD-fed *Ctrp1* KO mice had higher liver weight, hepatic cholesterol, and lower serum triglyceride and free fatty acid (FFA) [16]. However, *Ctrp1* KO mice showed different functional effects in HFD-fed conditions. Compared with controls, HFD-fed *Ctrp1* KO mice had less weight, lower liver weight, and down-regulation of lipid uptake and synthesis genes (*Scd1*, *Cd36,* and *PPARγ*) [16]. Mechanistic studies further indicated that CTRP1 had different functions in regulating the activity of AMPK among different nutrition conditions and tissues [16]. These results suggest that CTRP1 plays multiple roles in various tissues and pathological conditions, and the therapeutical role of CTRP1 in treating obesity, type II diabetes, and fatty liver remains elusive, as well as their underlying mechanisms.

In this study, we investigated the therapeutical effects and mechanisms of CTPR1 on diet-induced obesity, T2DM, and fatty liver. Overexpression of *CTRP1* by hydrodynamic gene delivery improved metabolic homeostasis in obese mice and STZ-induced diabetic mice, suppressed HFD-induced weight gain and the development of fatty liver, and alleviated insulin resistance. Our results indicate that human *CTRP1* gene delivery improves metabolic homeostasis and might act as a therapeutic target in obesity and T2DM.

## Materials and methods

### Plasmids

pLIVE^®^ Vector/SEAP Control Vector Kit (Cat. MIR 5620), including pLIVE and pLIVE-SEAP plasmids, were purchased from Mirus Bio (Madison, WI, USA). SEAP is secretory alkaline phosphatase, and pLIVE-SEAP plasmid was used as control. The human CTRP1 gene (NM_030968.5) was cloned and inserted into the pLIVE vector at *Nhe* I and *Xho* I sites (the map of the constructs was in Additional file 1: Figure S1). The constructed plasmid was confirmed by sequencing (Tsingke Biotechnology Co., Ltd., Beijing, China). The plasmid was extracted using the EndoFree Maxi Plasmid Kit (DP117, Tiangen, Beijing, China).

### Antibodies and reagents

Rabbit monoclonal antibody AKT (Cat. #4691, CST), p-AKT (Thr308, Cat. #13038, CST), AMPK (Cat. #5831, CST), p-AMPK (Thr172, Cat. #2535, CST), ERK1/2 (Cat. #4695, CST) and p-ERK1/2 (Thr202/Tyr204, Cat. #4376, CST) were supplied by Cell Signaling Technology (CST, Danvers, CO, USA). Mouse monoclonal antibody β-actin was punched from Proteintech (Cat. 66009–1-Ig, Chicago, IL, USA). Goat anti-rabbit IgG-HRP (Cat. ZB-2301) and goat anti-mouse IgG-HRP (Cat. ZB-2305) were supplied by ZSGB-BIO (Beijing, China). PrimeScript™RT Kit (Takara Bio, Dalian, China), QuantiFast SYBR Green PCR Kit (Qiagen, Duesseldorf, Germany), BCA Microalbumin Quantitation Kit for proteins (Applygen Technologies Inc., Beijing, China), human CTRP1 Elisa kit (Cat. ml038603-C, Mlbio, Shanghai, China), STZ (Cat. S8050, Solarbio, Beijing, China), Hematoxylin and eosin (H&E) staining kit (Cat. L11020102, Yulu experimental equipment Co., Ltd., Nanchang, China), human insulin (Humulin) (Eli Lilly, Indianapolis, IN, USA), the high-fat diet (60% kJ/fat, 20% kJ/carbohydrate, 20% kJ/protein, Cat. D12492, Research Diets Inc., New Brunswick, NJ, USA), and regular diet (Beijing Keao Xieli Feed Co., Ltd., Beijing, China) were supplied by specific companies.

### Animal experiment and procedure

C57BL/6 mice (male, ~ 25 g, ~ 8 weeks) were purchased from Changsha Tianqin Biotechnology Co. Ltd. (Changsha, China). The group-housed mice were kept at 25 ± 2 °C with a 12 h light–dark cycle. All animal protocol was approved by the Animal Ethics Committee of Nanchang University.

To investigate whether human *CTRP1* expression in mice could prevent HFD-induced obesity, mice were divided into three groups randomly (5 mice per group). All the mice were fed a regular chow diet for 2 weeks to adapt to the environment. After that period, mice in the CHOW group were continuingly fed the Chow diet. While mice in HFD/SEAP group and HFD/CTRP1 group were fed a high-fat diet. pLIVE-SEAP or pLIVE-CTRP1 plasmid DNA (1 μg/g body weight) was hydrodynamically injected into mice to give a high expression of the target gene in the liver (injected solution volume/body weight = 9%) according to the previous study [17]. The same amount of plasmid DNA was re-injected to keep the high gene expression 4 weeks after the first injection. The group was named HFD/SEAP group, HFD/CTRP1 group, and CHOW group. On days 1, 3, 7, 21, and 49, tail-tip blood was collected into 0.5 mL tubes containing sodium heparin. The plasma was extracted after centrifuging at 4 °C and 8000 rpm for 5 min. The weight and the food intake were measured per week. The blood glucose homeostasis was determined by the glucose tolerance test (GTT) and insulin tolerance test (ITT).

To investigate the effects of human CTRP1 expression on STZ-induced T2DM, C57BL/6 mice were randomly divided into two groups (8 mice in each group) and fed HFD for 40 days. Then, the mice were treated with STZ (50 μg/g body weight, dissolved in 0.1 M sodium citrate buffer solution) for 5 days. Mice were regarded as the diabetes model if blood glucose levels were higher than 13.9 mmol/L at least for two sequential days. Diabetic mice were injected with pLIVE-SEAP and pLIVE-CTRP1 plasmids, respectively, as described above. Mice were continually maintained on HFD for another 30 days. The non-fasting and fasting blood glucose were detected every 5 days, and GTT and ITT were performed by the end of the study.

In the diet-induced obese mouse model, mice were divided into two groups randomly and fed with HFD for 12 weeks, followed by hydrodynamically injecting with pLIVE-SEAP or pLIVE-CTRP1 plasmid DNA. The body weight and food intake were recorded. Blood glucose tests, GTT, and ITT were performed.

For the paired feeding experiment, mice were caged individually and randomly into two groups. pLIVE-SEAP and pLIVE-CTRP1 plasmid was hydrodynamically injected into these two groups, respectively. CTRP1 treated mice (HFD/CTRP1 group) were fed with HFD ad libitum. The control mice (treated with SEAP, HFD/SEAP) were pair-fed to match the food intake of HFD/CTRP1 mice (average amount of food consumed per day).

### GTT and ITT

In GTT, after 8 h of fasting, the blood glucose level was measured in mice using glucose testing strips and a blood glucose determinator (GA-3, Sannuo biosensor Co. Ltd.). Then the fasting mice were intraperitoneally injected with 20% body weight glucose, and the blood glucose was recorded for 30, 60, and 120 min. In ITT, mice fasted for 6 h, and blood glucose was measured using the same method as GTT. Then the mice were intraperitoneally injected with Humulin (0.75 U/kg of body weight). Blood glucose was recorded after 30, 60, and 120 min.

### Biochemical analysis

The tissue samples were weighed, digested by lysis solution, homogenized, and treated according to manufacturers’ instructions. Insulin was detected using ELISA Kit (Cat. ml001983, Mlbio, Shanghai, China). The commercial kits were used to detect serum aspartate aminotransferase (AST) and alanine aminotransferase (ALT) (Cat. AST03 and ALT03, Ningbo Purebio Biotechnology Co., Ltd., Ningbo, China), liver triglyceride (E1013, Applygen Technologies Inc.), total cholesterol (E1015, Applygen Technologies Inc.) and FFA (#15781, Diasy Diagnostic Systems, Frankfurt, Germany) following manufacturers’ instruction.

### Histochemical analysis

After being fixed with neutrally buffered 10% formalin for 24 h, the tissue samples, including livers, pancreas, and adipose tissues, were embedded with paraffin, cut into 5 μm sections, and stained by H&E. In the Oil-Red O staining experiment, the liver was embedded in OCT medium and frozen by liquid nitrogen. After being equilibrated in a cryostat, the samples were cut into 10 μm sections. The sections were fixed in neutrally buffered 10% formalin for 30 min, washed three times, stained in 0.5% Oil-Red O in 60% isopropanol for 5 min, counterstained by hematoxylin, washed, and sealed in neutral gum. All the sections were observed and captured with an optical microscope-equipped Nikon camera (Cat.DSU3, Nikon imaging instruments, Japan).

### Energy metabolism analysis

Mice were individually placed into an independent closed metabolic cage. After 1 day of adaption, the metabolism parameters were measured using Phenomaster (TSE System, Thuringia, Germany) according to the previous study [17]. Food and water consumption, animal activity, the O_2_ volume (VO_2_), and CO_2_ volume (VCO_2_) were recorded. The calculation of VO_2_, VCO_2_, respiratory exchange ratio (RER), and energy expenditure (EE) referred to our previous study [17]. In brief, EE was calculated as EE = 3.941 × VO_2_ + 1.106 × VCO_2_, and RER was calculated as RER = VCO_2_ [mL/h/kg]/VO_2_ [mL/h/kg].

### Quantitative real-time PCR

Total RNA of the liver, WAT, and BAT was extracted using TRIzol reagent (Invitrogen, Carlsbad, CA, USA). PrimeScript^™^RT (Takara Bio) was used for reverse transcription. SYBR-Green PCR Kit (Qiagen) was used for qPCR. GAPDH and β-actin were used as internal references. The absence of non-specific binding of primers was verified by blasting. The sequence of primers, GenBank Accession numbers, expected size of the amplicon, and annealing temperatures were listed in the Additional file 1: Table S1.

### Western blotting

The total protein of the liver was extracted using RIPA buffer to extract the total protein, and adipose tissue total protein was extracted by kit (Cat. BB312262, BestBio, Shanghai, China). BCA kit was used to measure the concentration of protein samples. Then the sample was mixed with 5 × loading buffer (volume ratio = 4:1), incubated at 100 °C for 5 min, and stored at −20 °C. Equal protein quantities (100 μg) were loaded and electrophoresed by SDS-PAGE. Then the proteins were transferred from the gel to the Immobilon-P PVDF membrane (Millipore Corp, Billerica, MA, USA) using Mini-PROTEAN^®^Tetra System (Bio-Rad, Hercules, CA, USA) at 200 mA for 90 min. The membrane was washed and incubated in 5% non-fat milk in 1 × TBST buffer for 1 h and incubated with the primary antibody overnight at 4 °C (the dilution ratio is according to manufacturers’ instructions). Then the membrane was washed with 1 × TBST buffer three times and incubated with secondary antibody (the dilution ratio is according to manufacturers’ instructions) for 2 h at room temperature. After washing, the membrane was detected using a chemiluminescence imager (3600 Mini, Shanghai Qinxiang Scientific Instrument Co. Ltd, Shanghai, China). β-actin was used as an internal reference, and the protein level of a specific phosphorylated protein was normalized by its total protein.

### Statistical analysis

The data were analyzed using Student’s *t*-test or one-way ANOVA. The data were analyzed using the Shapiro–Wilk test and Levene’s test for their normality and homogeneity. Post-hoc comparisons were performed using LSD and SNK tests in one-way ANOVA. If the logarithmically transformed data did not conform to a normal distribution, Kruskal-WallisH and Mann–Whitney U tests were used. The data were expressed as mean ± SEM. *P* < 0.05 was considered significant difference.

## Result

### Overexpression of CTRP1 prevented HFD-induced body weight gain

**Fig. 1:**
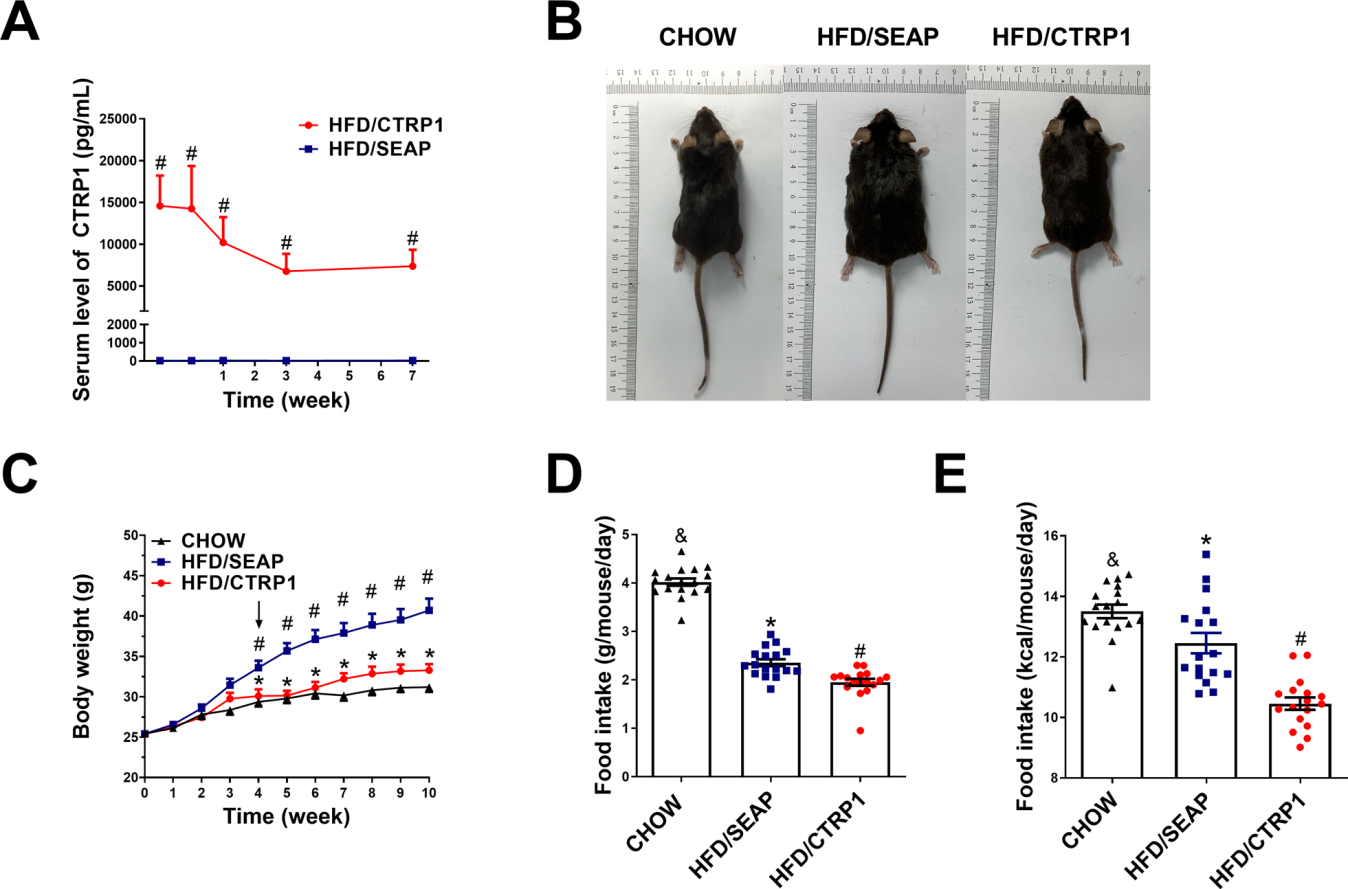
Hydrodynamic gene transfer of *CTRP1* prevents HFD-induced obesity. **A**. The concentrations of CTRP1 protein in blood after plasmid injection; **B**. Representative pictures of mice at the end of the experiment; **C**. Mouse growth curve; **D**. Food intake; **E**. kcal of food intake in mice. *significant difference between HFD/SEAP group and Chow group (*P* < 0.05), #significant difference between HFD/CTRP1 group and HFD/SEAP group (*P* < 0.05), & significant difference between HFD/CTRP1 group and CHOW group (*P* < 0.05)

*CTRP1* overexpressed mice model was established by hydrodynamic injection according to the previous study [17]. The serum CTRP1 protein level peaked at 48 h (~ 15000 pg/mL) and was sustained for more than 8 weeks (Fig. 1A). CTRP1 protein level was kept at 8000 pg/mL at the end of the experiment, which was approximately 40-fold that of the control group (200 pg/mL, secreted SEAP gene was delivered into mice as the control). CCK8 assay showed no significant cytotoxicity in HepG2 cells after CTRP1 treatment (Additional file 1: Figure S2). Serum AST and ALT had no significant change (Additional file 1: Figure S3A), indicating that overexpression of *CTRP1* did not cause liver damage.

The exogenous expression of *CTRP1* prevented HFD-induced body weight gain (Fig. 1B, C). At the 10th week of injection, the average body weight of mice in the HFD-fed control group (HFD/SEAP) was 40 g, which was significantly higher than mice in HFD/CTRP1 group (~ 33 g). A significant difference could also be observed in the body size (Fig. 1B). The average food intake and calorie intake of mice in the HFD/CTRP1 group were significantly lower than that in HFD/SEAP group (Fig. 1D, E, Additional file 1: Figure S3B, C).

### CTRP1 treatment prevented HFD-induced adipocyte hypertrophy

**Fig. 2:**
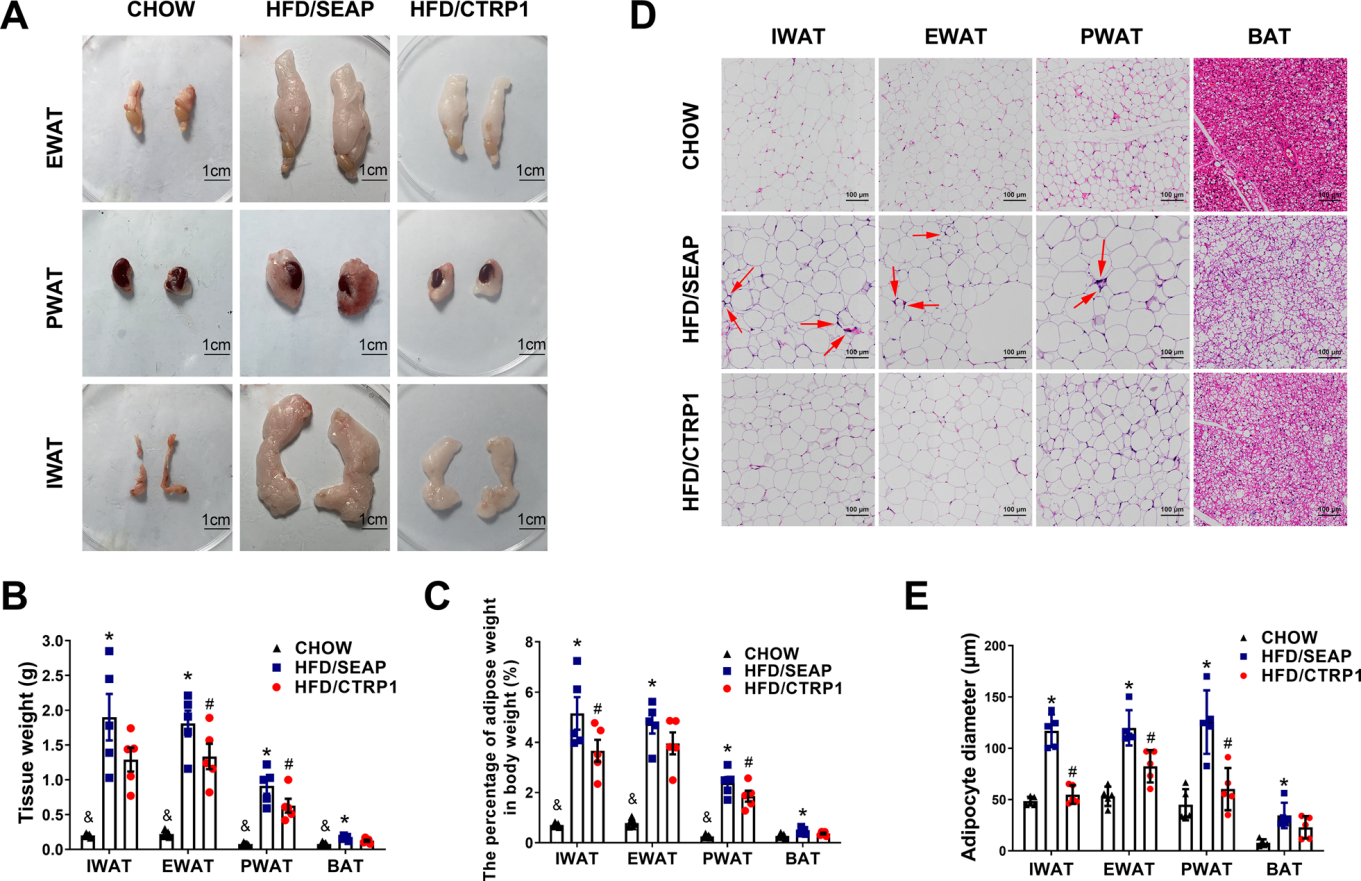
CTRP1 inhibits adipose hypertrophy. **A**. The morphology of white epididymal fat (EWAT), white perirenal fat (PWAT), and white inguinal fat (IWAT); **B**. Adipose tissue weight; **C**. The percentage of adipose tissue weight in the body weight; **D**. H&E staining. Arrows point to crown-like structures; **E**. Diameters of adipocyte. The data were analyzed by one-way ANOVA and presented by mean ± SEM (*n* = 5). *significant difference between HFD/SEAP group and CHOW group (*P* < 0.05), #significant difference between HFD/CTRP1 group and HFD/SEAP group (*P* < 0.05), & significant difference between HFD/CTRP1 group and CHOW group (*P* < 0.05)

To investigate the impact of CTRP1 on adipose tissue, different adipose tissues were collected at the end of the animal experiment. Overexpression of *CTRP1* significantly decreased the size and weight of white adipose tissue (WAT) and brown adipose tissue (BAT) (Fig. 2A–C, Additional file 1: Figure S4). Less accumulation of epididymal WAT (EWAT), perirenal WAT (PWAT), and inguinal WAT (IWAT) were found in the CTRP1 treated group compared to that in the HFD-fed control group (Fig. 2A). H&E staining of WAT showed that HFD significantly induced adipocyte hypertrophy, and the size of adipocytes in WAT was significantly smaller in HFD/CTRP1 group than that in HFD/SEAP group (Fig. 2D, E, Additional file 1: Figure S4). Similarly, CTRP1 treatment mice also showed fewer lipids accumulation in BAT (Fig. 2D, E, Additional file 1: Figure S4). These results indicate that overexpression of *CTRP1* prevents HFD-induced adipocyte hypertrophy.

### CTRP1 treatment prevented the development of HFD-induced fatty liver

**Fig. 3:**
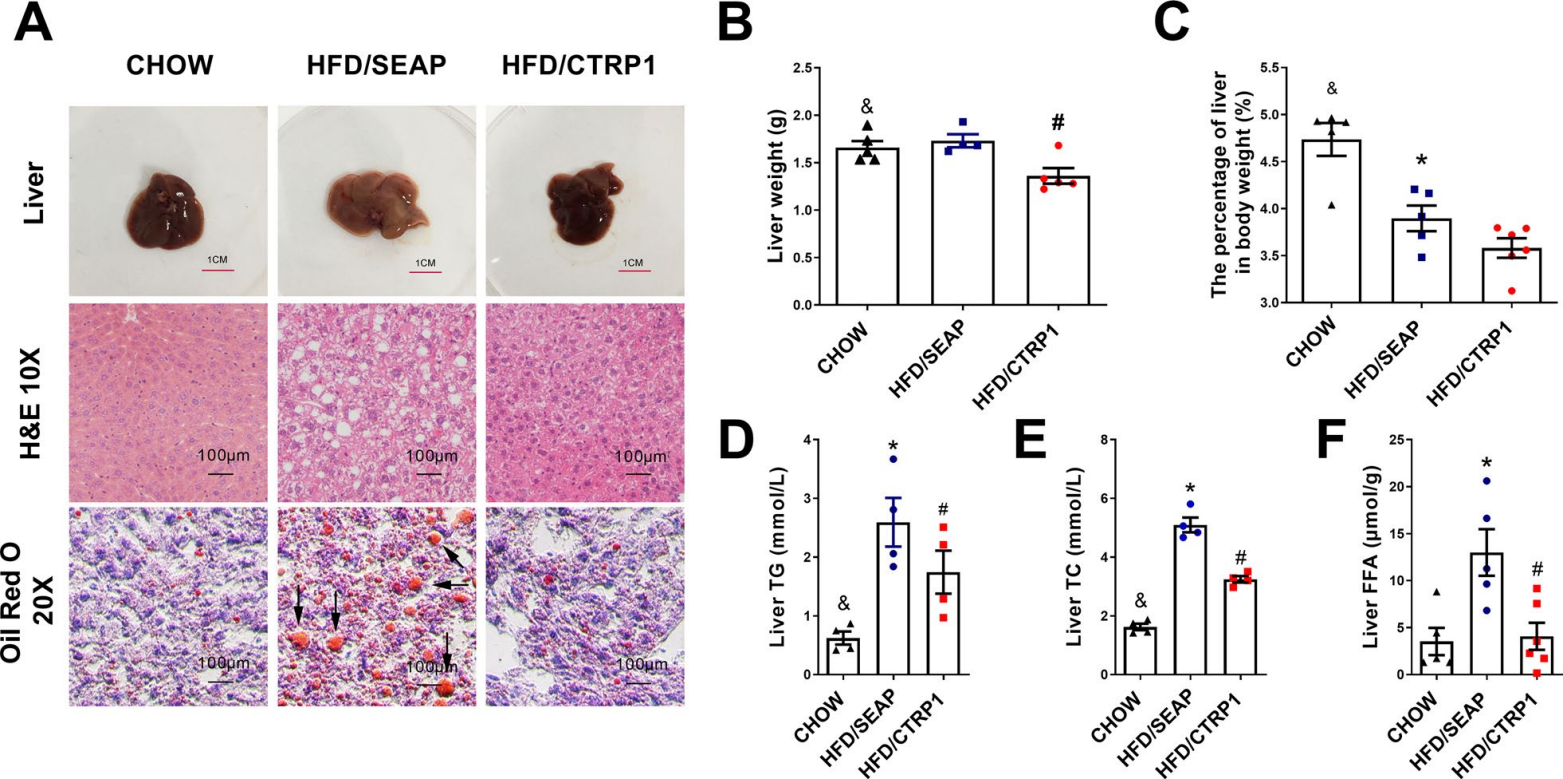
CTRP1 inhibits HFD-induced fatty liver. **A**. The representative pictures, H&E staining, and oil red O staining of the liver. Arrows point to large lipid droplet; **B**. The weight of the liver; **C**. The percentage of liver weight in the body weight of mice; **D**. Triglyceride in the liver; **E**. Cholesterol in the liver; **F**. Fatty acid in the liver. The data were analyzed by one-way ANOVA and presented by mean ± SEM (*n* = 5). *significant difference between HFD/SEAP group and CHOW group (*P* < 0.05), #significant difference between HFD/CTRP1 group and HFD/SEAP group (*P* < 0.05), & significant difference between HFD/CTRP1 group and CHOW group (*P* < 0.05)

Obesity is usually related to the over-accumulation of fat in the liver. The liver of HFD/CTRP1 mice had a smaller size and less weight than the HFD control group (Fig. 3A–C). H&E staining and Oil-Red O staining showed that *CTRP1* gene delivery inhibited the enrichment and enlargement of lipid droplets (LDs) (Fig. 3A, Additional file 1: Figure S5). The concentrations of liver triacylglycerols (TG), total cholesterol (TC), and free fatty acid (FFA) were significantly lower in CTRP1-overexpressed mice than that in the HFD-fed control (Fig. 3D–F). These results demonstrate that *CTRP1* gene delivery prevents HFD-induced occurrence of fatty liver.

### CTRP1 treatment prevented HFD-induced glucose intolerance and insulin resistance

**Fig. 4:**
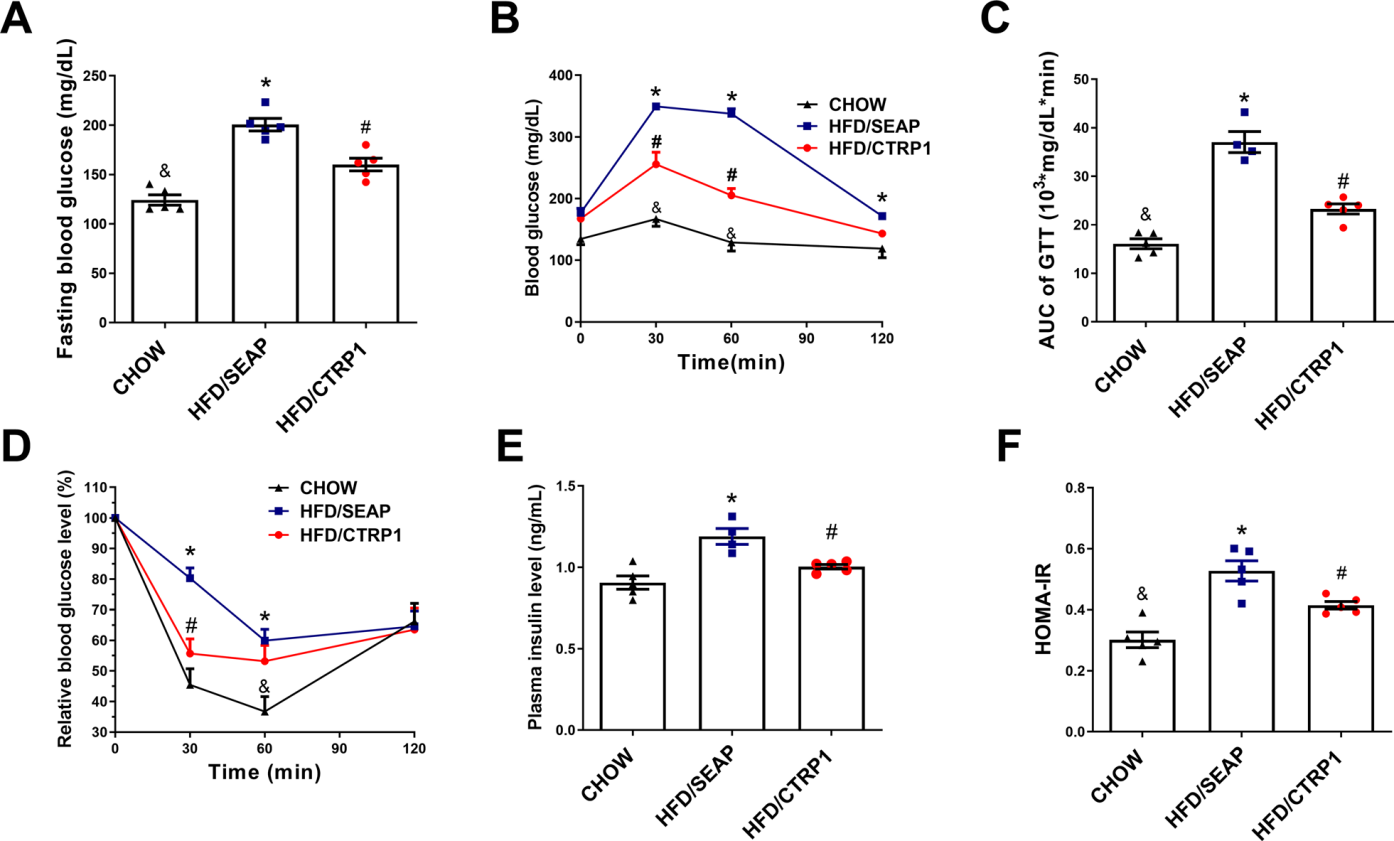
CTRP1 improves glucose homeostasis in HFD-fed mice. **A**. Fasting blood glucose; **B**. Glucose tolerance test; **C**. Area under the curve of glucose tolerance test; **D**. Insulin tolerance test; **E**. The concentration of insulin in the plasma of mice; **F**. The insulin resistance index. The data were presented by mean ± SEM (*n* = 5). *significant difference between HFD/SEAP group and CHOW group (*P* < 0.05), #significant difference between HFD/CTRP1 group and HFD/SEAP group (*P* < 0.05), & significant difference between HFD/CTRP1 group and CHOW group (*P* < 0.05)

HFD-induced obesity usually disrupts glucose homeostasis. To examine the effect of CTRP1 on glucose homeostasis in HFD-fed mice, GTT and ITT were performed. Fasting blood glucose in HFD/CTRP1 group was significantly lower than that in the HFD control group (Fig. 4A). GTT assay and calculated area under the curve (AUC) demonstrated that CTRP1 improved glucose clearance (Fig. 4B, C). In the ITT assay, CTRP1-treated mice were more sensitive to insulin injection than the HFD control mice on weeks 8 and 12 (Fig. 4D, Additional file 1: Figure S6). CTRP1 did not significantly affect the insulin secretion of mouse islets in vitro after glucose challenge (Additional file 1: Figure S7). The plasma insulin level of mice in the HFD/CTRP1 group was lower than that in HFD/SEAP group (Fig. 4E), which demonstrated that CTRP1 improved insulin sensitivity. HOMA-IR also showed that CTRP1 prevented the development of insulin resistance (Fig. 4F). These results indicate that CTRP1 treatment inhibited HFD-induced glucose intolerance and insulin resistance.

### CTRP1 improved glucose homeostasis in STZ-induced type II diabetes mice

**Fig. 5:**
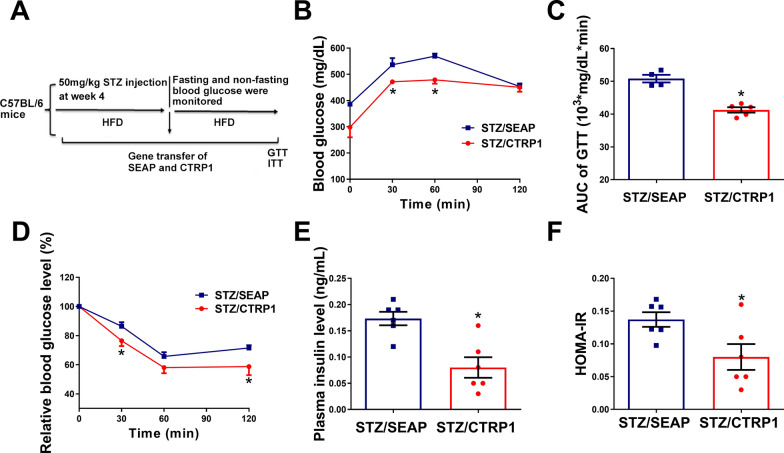
Effect of *CTRP1* gene transfer on type II diabetes. **A**. the flow chart of animal experiment **B**. Glucose tolerance test; **C**. The area under curve of GTT; **D**. Insulin tolerance test; **E**. The concentration of insulin in plasma; **F**. Insulin resistance index. The data were analyzed by *t*-test; the data were presented by mean ± SEM (STZ/SEAP, *n* = 8, STZ/CTRP1, *n* = 9), *significant difference (*P* < 0.05)

To further investigate the effect of CTRP1 in T2DM, the STZ-induced T2DM mice model was established and treated with CTRP1 (Fig. 5A). GTT assay showed that overexpression of CTRP1 improved glucose intolerance in STZ-induced T2DM mice (Fig. 5B, C). ITT assay demonstrated that CTRP1-treated mice were more sensitive to insulin than control mice (Fig. 5D). The concentration of plasma insulin in the STZ/CTRP1 group was significantly lower than that in STZ/SEAP group (Fig. 5E). Correspondingly, HOMA-IR also showed that insulin sensitivity of STZ/CTRP1 mice was improved compared with the control group (Fig. 5F). The body weight of CTRP1-treated mice (STZ/CTRP1 group) was slightly lighter than that of the control group (STZ/SEAP) but without a statistical difference (Additional file 1: Figure S8A). The food intake and fasting blood glucose of mice showed no statistical significance between CTRP1 treated and control groups (Additional file 1: Figure S8B, C). These results indicate that CTRP1 improves glucose homeostasis in STZ-induced T2DM.

### CTRP1 improved glucose homeostasis in obese mice

**Fig. 6:**
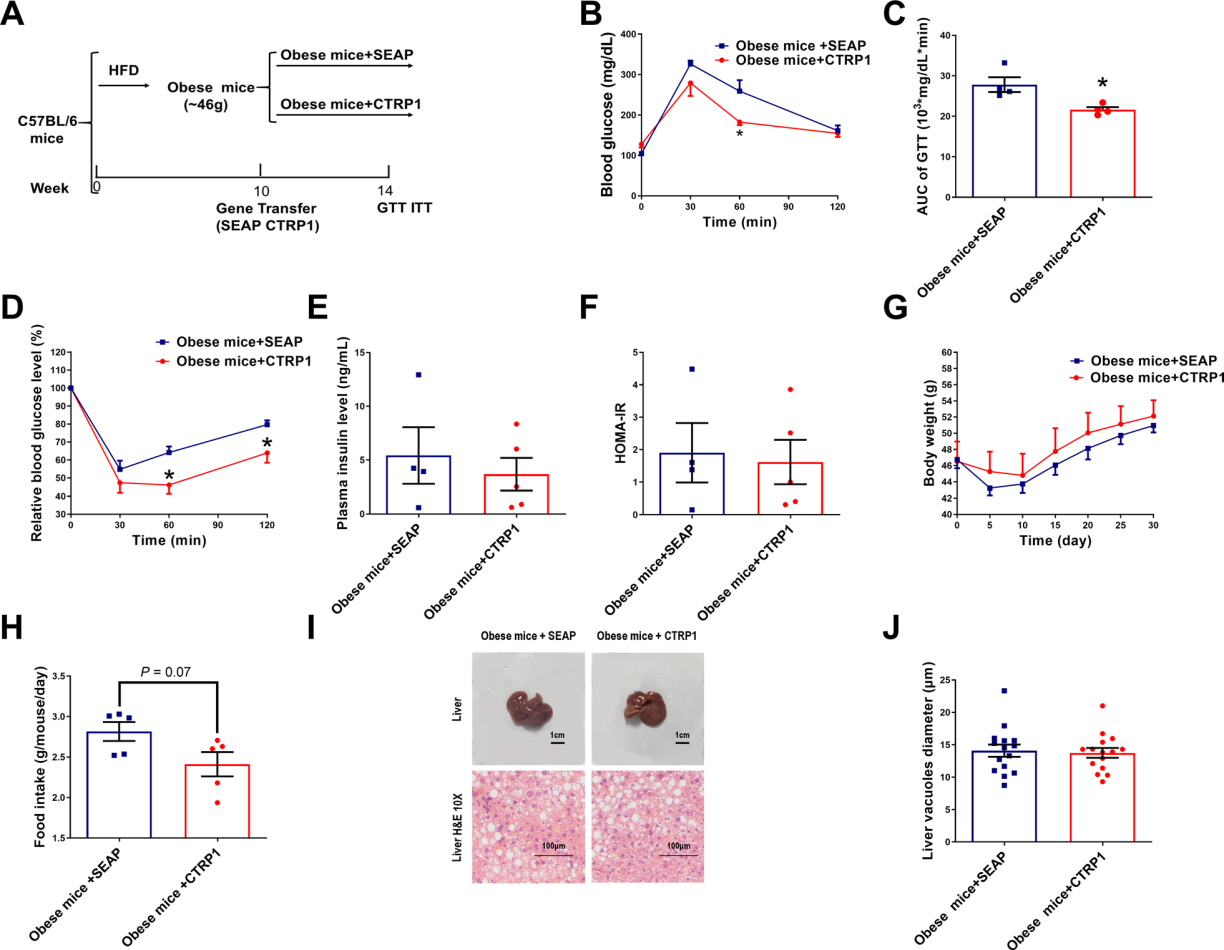
The effect of CTRP1 in the obese mouse model. **A**. Flow chart of the experiment; **B**. Glucose tolerance test; **C**. The area under curve of GTT; **D**. Insulin tolerance test; **E**. The concentration of insulin in mice plasma; **F**. Insulin resistance index; **G**. The curve of body weight change; **H**. Food intake; **I**. Representative pictures of the liver and H&E staining; **J**. Statistics of fat vacuole diameter in liver H&E staining. The data were analyzed by *t*-test and presented by mean ± SEM (*n* = 5), *significant difference (*P* < 0.05)

Based on the therapeutic effects of CTRP1 on HFD-induced obesity and STZ-induced T2DM mice, the obese mouse model was established to investigate whether CTRP1 can improve glucose metabolism and decrease body weight. After 12 week HFD feeding, the average body weight of mice was more than 46 g, indicating the successful establishment of the obese mouse model. Then the mice were treated by hydrodynamic injection of *CTRP1* (Fig. 6A). To test the effect of CTRP1 on glucose metabolism, GTT and ITT assays were performed after a 4 week CTRP1 treatment. In the GTT assay, CTRP1-treated mice showed a higher glucose clearance rate than control mice (Fig. 6B). The AUC of GTT also confirmed that CTRP1 improved glucose tolerance (Fig. 6C). ITT assay showed that CTRP1 increased the insulin sensitivity of obese mice (Fig. 6D). However, the fasting plasma insulin levels and HOMA-IR were not significant between CTRP1 treated and control mice (Fig. 6E, F). Overexpression of *CTRP1* had no significant effects on the body weight of obese mice (Fig. 6G), as well as the weight of liver and adipose tissues. CTRP1 treatment slightly decreased the food intake (*P* = 0.07) (Fig. 6H). H&E staining showed that the size of liver adipose droplets and vacuoles were also similar between these two groups (Fig. 6I, J). These results indicate that CTRP1 improves glucose tolerance and insulin sensitivity in obese mice. However, CTRP1 could not decrease body weight and hepatic steatosis in obese mice.

### CTRP1 enhanced oxygen consumption and energy expenditure

**Fig. 7:**
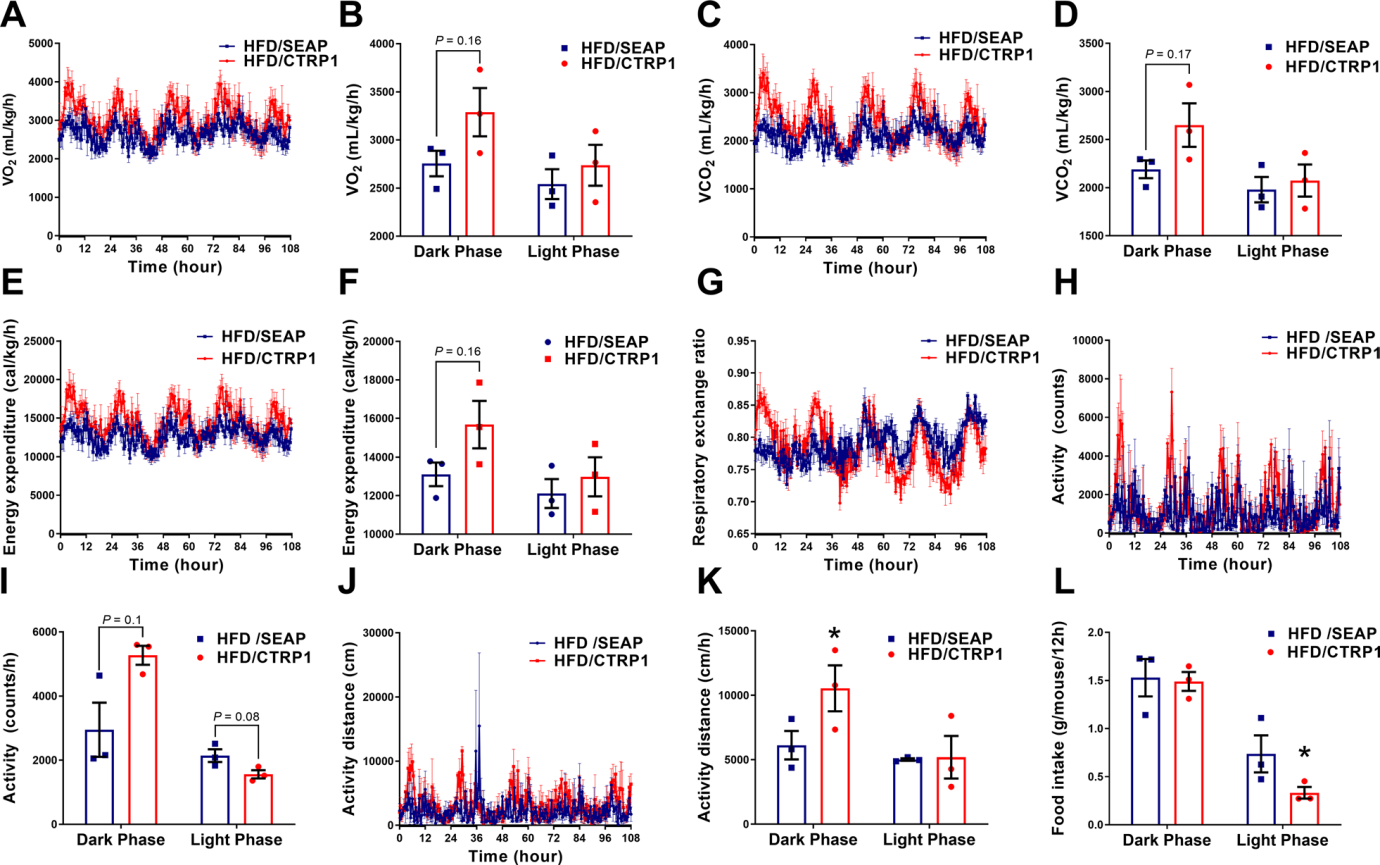
Metabolic cage analysis. **A**. Oxygen consumption of mice; **B**. Statistical analysis of VO_2_ in dark and light phase; **C**. Carbon dioxide exhalation; **D**. Statistical analysis of VCO_2_ in dark and light phase; **E**. Energy expenditure; **F**. Statistical analysis of energy expenditure in dark and light phase; **G**. RER; **H**. Activity counts; **I**. Statistical analysis of the activity counts in dark and light phase; **J**. Activity distance; **K**. Statistical analysis of the activity distance in dark and light phase; **L**. Food intake. The dark and light phases were indicated by black and white bars on the x-axis. The data were displayed by mean ± SEM (*n* = 3), *significant difference (*P* < 0.05)

A metabolic cage assay was performed to investigate the mechanism underlying the preventing role of CTRP1 in HFD-induced weight gain and metabolic disorders. Compared to mice in HFD/SEAP group, CTRP1-treated mice showed more oxygen consumption in the dark phase (VO_2_ and VCO_2_, *P* = 0.16 and 0.17, respectively) (Fig. 7A–D). CTRP1 treatment also increased mice's energy expenditure in the dark phase (*P* = 0.16) (Fig. 7E, F). RER of mice showed no significant difference in the two groups (Fig. 7G). The activities assay showed that CTRP1-treated mice were more active in the dark phase than control mice (Fig. 7H–K). The food intake in CTRP1-treated mice was less than that in control mice in the light phase, but there was no significant difference in the dark phase (Fig. 7L). These results indicate that CTRP1 enhances oxygen consumption and energy expenditure.

### CTRP1 decreased the food intake of mice and upregulated leptin

**Fig. 8:**
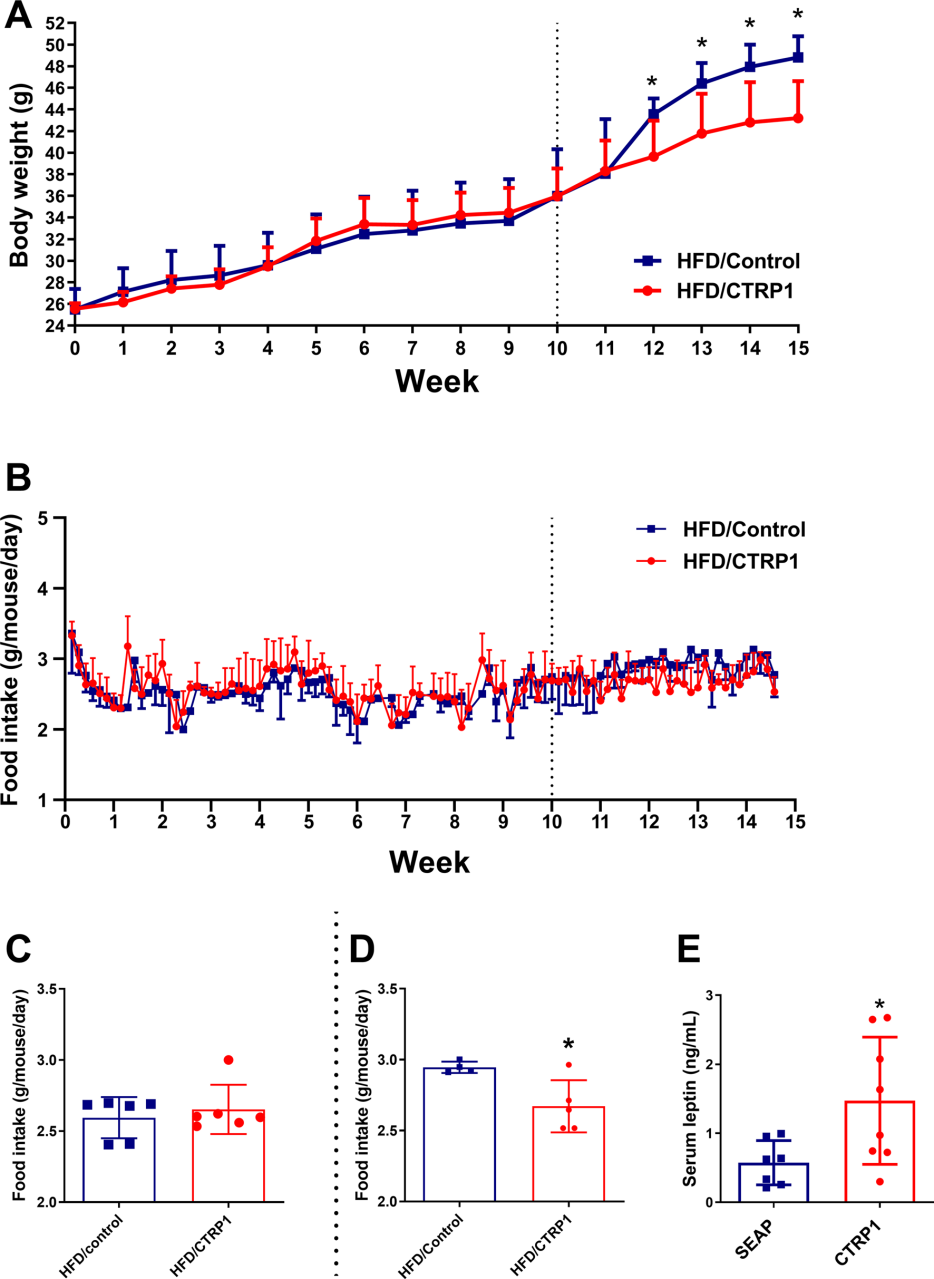
Paired feeding experiment and the effects of CTRP1 on leptin. **A**. The body weight curve. Left of the dotted line, paired feeding (control mice were restricted to the average amount of food intake of CTRP1 treated mice); right of the dotted line, ad libitum fed for both groups; **B**. Food intake curve; **C**. Average food intake during paired feeding; **D**. Average food intake after withdrawing paired feeding; **E**. the concentration of leptin in the blood. The data were analyzed by t-test and presented by mean ± SEM (*n* = 5). *significant difference (*P* < 0.05)

The metabolic cage assay and the HFD-induced obesity model showed that CTRP1 decreased the food intake of mice. To confirm the effects of CTRP1 on food intake and appetite, we performed a paired feeding experiment. The CTRP1 treated mice were ad libitum fed with HFD, while the control group was restricted fed with the same amount of food given to CTRP1 treated mice for 10 weeks. The body weight showed no difference between the HFD/SEAP group and the HFD/CTRP1 group during the period of paired feeding (Fig. 8A–C). When the food restriction was withdrawn at week 10, the food intake of the HFD/SEAP group was significantly increased and higher than that of the HFD/CTRP1 group (Fig. 8A, B, D). This result indicated that CTRP1 prevented body weight gain by decreasing food intake. To investigate the underlying mechanism, the serum leptin level was tested. The leptin level in the blood circulation of CTRP1-treated mice was significantly increased compared with the control group (Fig. 8E). The mRNA levels of appetite-related genes in the hypothalamus and intestine were also detected. The expression of *Agrp* was down-regulated, but no significant difference was observed (*P* = 0.16, Additional file 1: Figure S9). These results suggested that CTRP1 inhibited food intake and appetite by upregulating the level of leptin.

### CTRP1 changes the expression of glucose and lipid metabolism-related genes

**Fig. 9:**
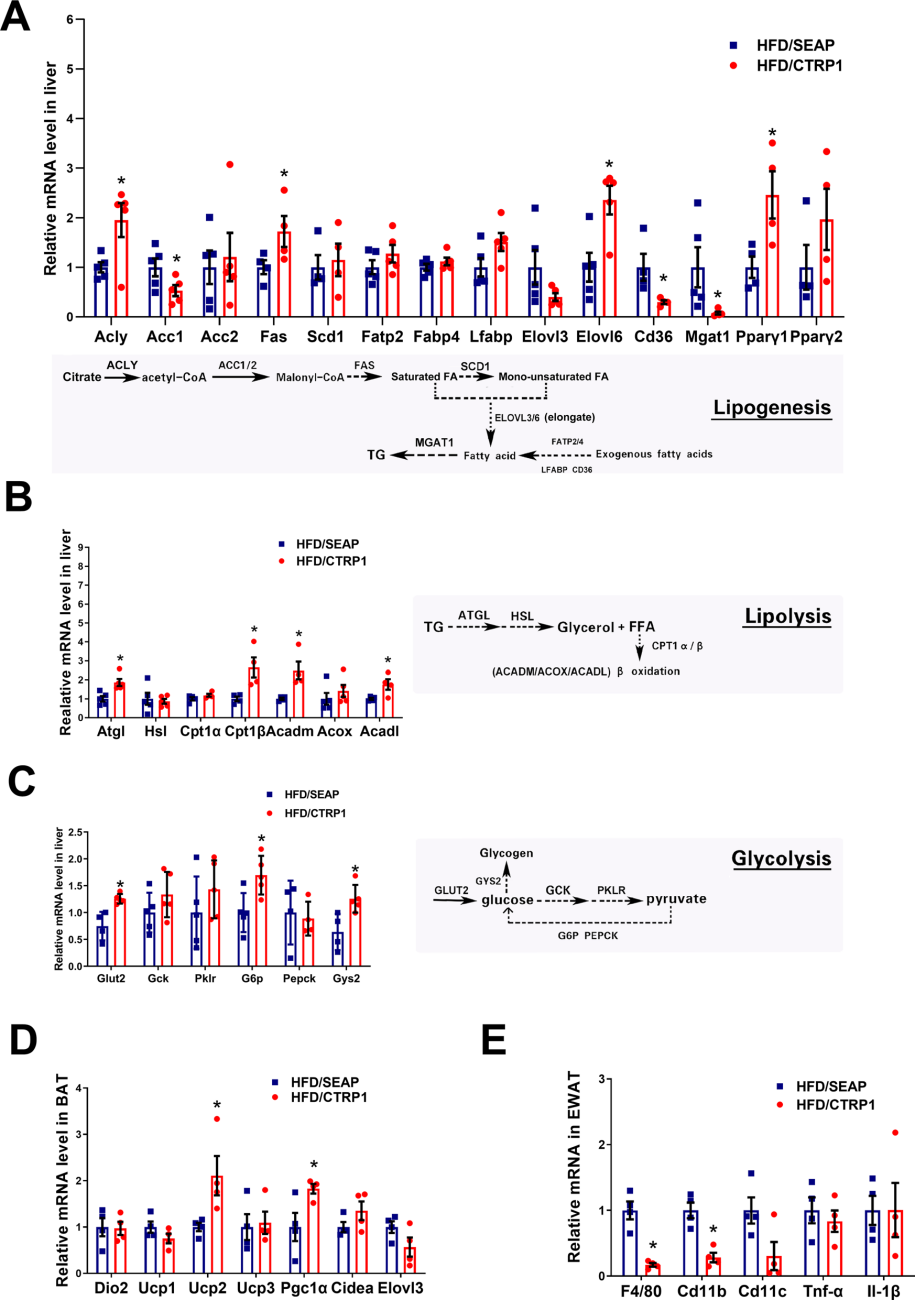
The regulation of hydrodynamic gene transfer *CTRP1* on related genes in liver and adipose tissue in high-fat diet-induced obesity model. **A**. The expression of lipogenesis genes in the liver; **B**. The expression of lipolysis and β oxidation-related genes in the liver; **C**. The expression of glycogen synthesis genes in the liver; **D**. The expression of thermogenesis genes in BAT; **E**. The expression level of inflammation genes in EWAT. The data were analyzed by one-way ANOVA and compared in pairs. The data were displayed by mean ± SEM (*n* = 5), *P < 0.05

The expression of glucose and lipid metabolism-related genes was determined to find the underlying mechanisms of CTRP1 in preventing HFD-induced obesity and metabolic disorders. Compared to the HFD control group, *CTRP1* gene transfer significantly down-regulated the expression of some lipogenic genes, including *Acc1*, *Cd36,* and *Mgat1* (Fig. 9A). CTRP1 treatment increased the expression of lipolysis and β oxidation-related genes, including *Atgl*, *Cpt1β, Acadl,* and *Acadm* (Fig. 9B). It was also observed that some genes were up-regulated, which are responsible for glucose uptake, gluconeogenesis, glycogen synthesis, and lipogeneses, such as *Glut2*, *G6p, Gys2, Acly*, *Fas*, *Elovl6,* and *Pparγ1* (Fig. 9A–C). These results indicate that lipogenesis, lipolysis, and glycolysis are involved in the glucose and lipid homeostasis of HFD-fed mice after CTRP1 treatment.

### CTRP1 regulated the genes expression of inflammation and thermogenesis

The upregulation of oxygen consumption and energy expenditure in CTRP1 treated mice suggested the role of CTRP1 in regulating thermogenesis. The effect of CTRP1 on thermogenesis was investigated by detecting the expression of thermogenesis-related genes. *Pgc1α* and *Ucp2* in BAT of *CTRP1*-treated mice were significantly up-regulated compared with control mice (Fig. 9D), suggesting that CTRP1 increased the thermogenesis of mice.

Inflammation also plays an important role in HFD-induced obesity and metabolic disorder. Results from the qPCR analysis showed that inflammatory genes *F4/80*, *Cd11b,* and *Cd11c* were significantly inhibited in the WAT of CTRP1/HFD mice, compared with that in the control group (Fig. 9E), while *Tnf-α* and *Il-1β* showed no significance between these two groups (Fig. 9E). These data indicate that CTRP1 prevents HFD-induced inflammation in white adipose tissue.

### CTRP1 activated AMPK, PI3K/AKT, and ERK pathway in HFD mice

**Fig. 10:**
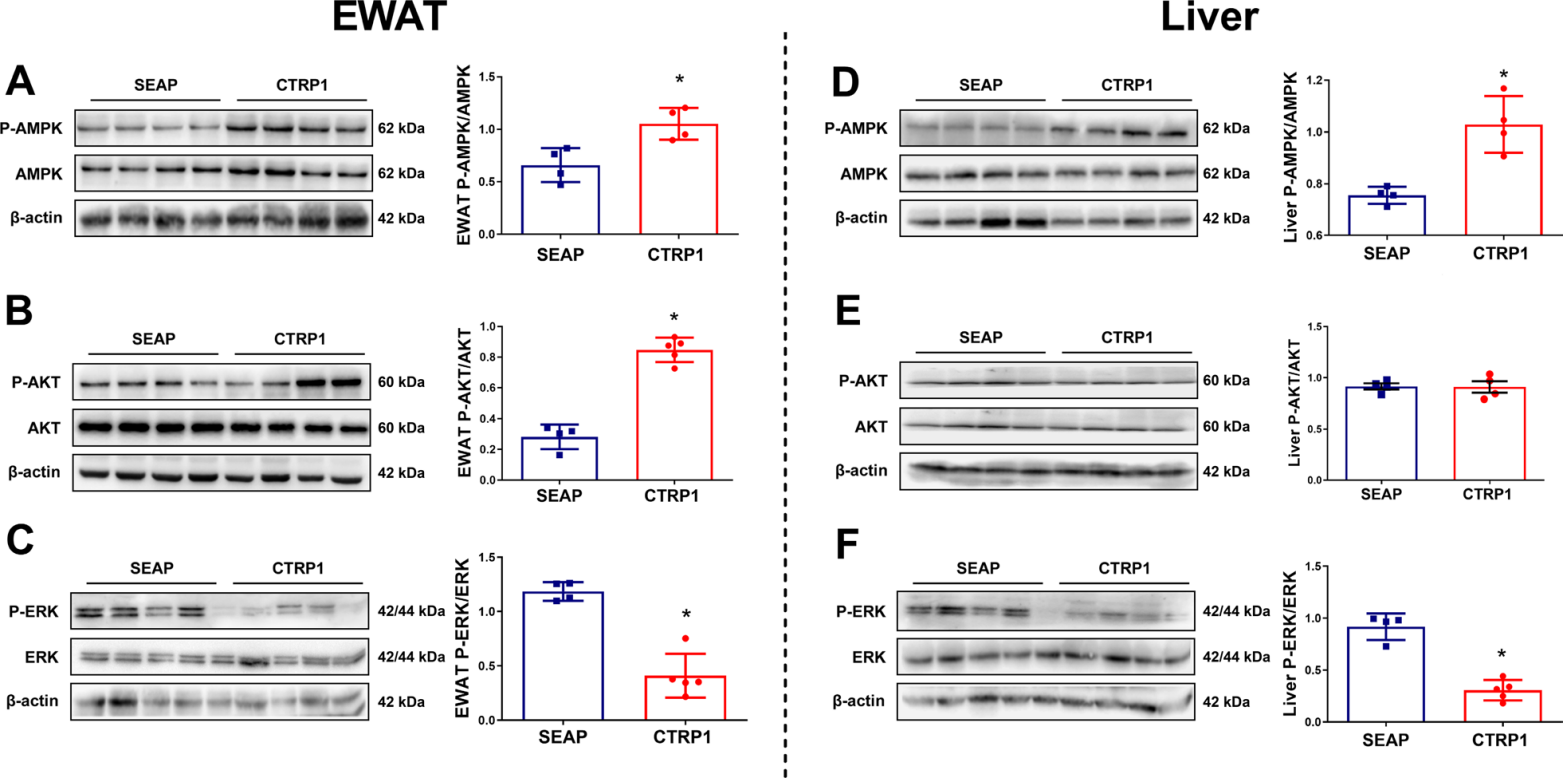
The regulation of AKT, AMPK, and ERK by CTRP1 in the liver and white adipose tissue. **A**. Western blotting of AMPK and P-AMPK in EWAT; **B**. Western blotting analysis of AKT and P-AKT in EWAT; **C**. Western blotting analysis of ERK and P-ERK in EWAT; **D**. Western blotting analysis of AMPK and P-AMPK in the liver; **E**. Western blotting analysis of AKT and P-AKT in the liver; **F**. Western blotting analysis of ERK and P-ERK in the liver. The data were analyzed by *t*-test and presented by mean ± SEM (*n* = 4), *P < 0.05

Metabolic disturbances may attribute to the disruption of multiple important signaling pathways, including energy sensor and metabolic center pathway—AMPK pathway [18], important insulin sensitivity and metabolism-related pathway—Akt pathway [19], and glucose metabolism-related pathway—ERK pathway [20]. Results in Western Blot analysis showed that the phosphorylation level of AMPK was upregulated in the EWAT and liver of CTRP1 treated mice compared with control mice (Fig. 10A, D). The expression of AMPK upstream genes LKB1 in the HFD/CTRP1 group was also slightly up-regulated compared with the control group (*P* = 0.2) (Additional file 1: Figure S10). *CTRP1* gene transfer increased the phosphorylation level of AKT in EWAT (Fig. 10B), while no significant difference was observed in the liver (Fig. 10E). CTRP1 treatment decreased the phosphorylation of ERK in EWAT and the liver (Fig. 10C, F). These results suggest that CTRP could regulate glucose and lipid metabolism in the liver and EWAT by modulating the AMPK, AKT, and ERK pathways.

## Discussion

**Fig. 11:**
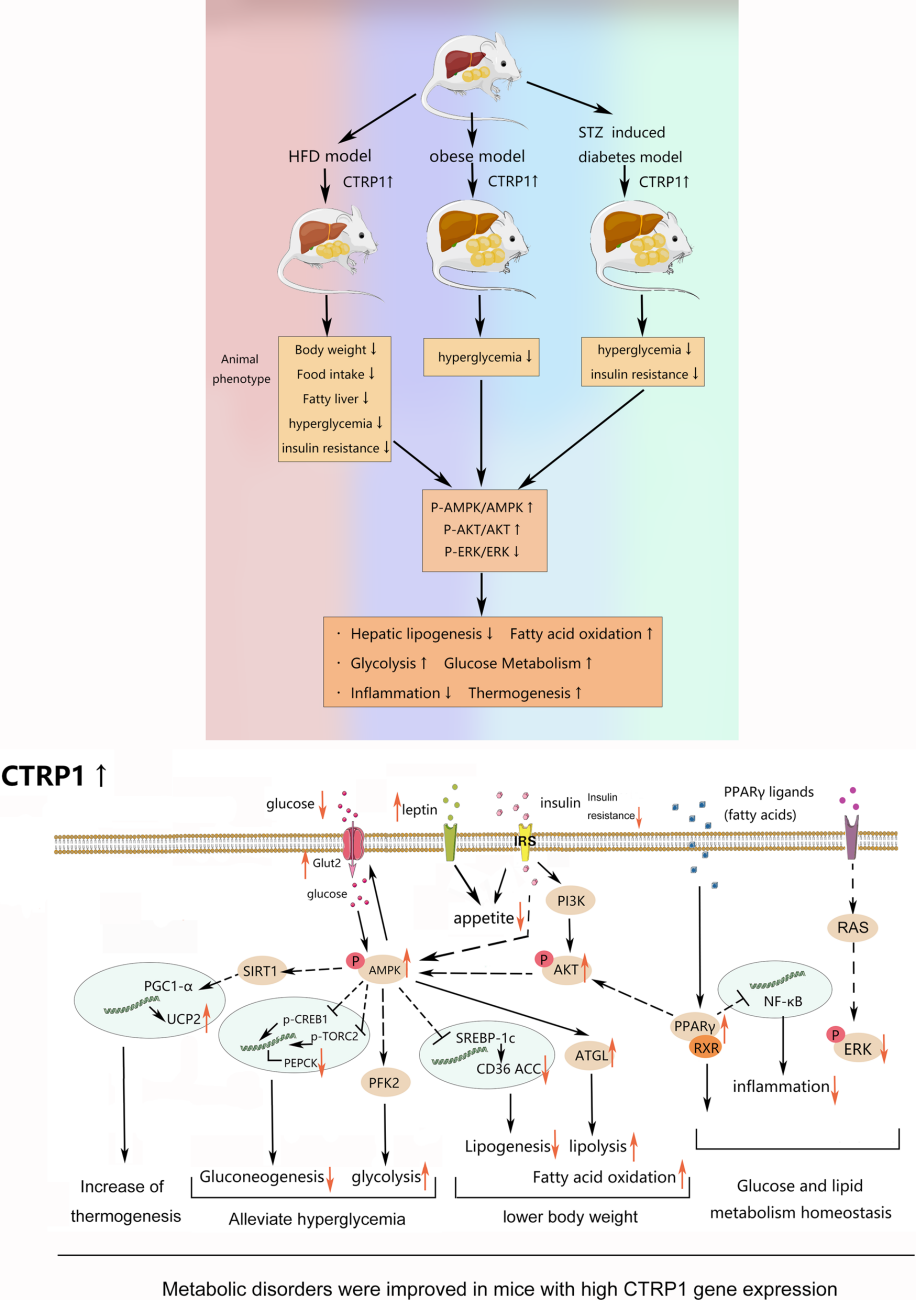
Summary of the beneficial effects of CTRP1 and signaling pathways

In this study, we demonstrated the therapeutic effects of CTRP1 in improving glucose homeostasis and preventing HFD-induced obesity, hyperglycemia, insulin resistance, and fatty liver in multiple metabolic disorder models. The increase in energy expenditure and reduced caloric intake may be involved in the physiological effects of CTRP1. Mechanistically, CTRP1 increased the protein level of leptin, upregulated the gene expression involved in thermogenesis, lipolysis, and glycolysis, and downregulated the expression of inflammatory genes. Moreover, CTRP1 activated AMPK and PI3K/Akt signaling pathways and inhibited ERK signaling pathways (Fig. 11). These results imply the important therapeutic role of CTRP1 and the potential to serve as a target in treating metabolic diseases.

Consistent with our findings, previous studies also verify the beneficial effects of CTRP1 in improving glucose homeostasis. *Ctrp1* transgenic mice showed better glucose homeostasis, and *Ctrp1* KO mice showed increased gluconeogenesis and insulin resistance in LFD-fed mice [16]. CTRP1 recombination protein also decreased blood glucose in wild mice [5]. Multiple mechanisms might be involved. Firstly, CTRP1 may improve glucose homeostasis through AKT signaling pathways. CTRP1 activated AKT signaling pathways in muscle myoblast C2C12 cells [5, 14], which increased glucose uptake and glycolysis. A clinical study showed that CTRP1 could inhibit the phosphorylation of IRS-1, a well-known upstream protein of AKT, to improve insulin resistance [15]. Our result also demonstrated that CTRP1 enhanced the phosphorylation of AKT in EWAT (Fig. 10B). Overexpression of CTRP1 increased the glucose uptake gene *Glut2*, glycogenic gene *Gys2* (Fig. 9C), suggesting the enhancement of insulin sensitivity and glucose metabolism after CTRP1 treatment. Secondly, CTRP1 might treat metabolic disorders by inhibiting the activation of macrophages and inflammation [21]. Previous studies showed that LPS (a proinflammatory substance) and oxidized LDL (oxLDL) could up-regulate the expression of *Ctrp1* in mice [6, 21]. CTRP1 inhibited the expression of *IL-6* and *IL-1β* and phosphorylation of NF-κB via the SP1/cAMP axis [22]. Knockdown of CTRP1 increased the protein level of TNF-α, NF-KB, and IL-1β induced by oxygen and glucose deprivation and reperfusion, and recombinant CTRP1 inhibited this induction [23]. The conclusions of the previous evidence are consistent with our results that CTRP1 significantly down-regulated the expression of inflammatory genes *Cd11b* and *Cd11c* in WAT and macrophage marker gene *F4/80* (Fig. 9E). The development of crown-like structures in white adipose tissue was also blocked by CTRP1 treatment (Fig. 2D), indicating the blockage of macrophage infiltration and migration to WAT. As inflammation plays an important role in obesity and multiple metabolic complications, the anti-inflammation functions of CTRP1 may contribute to the beneficial effects of CTRP1 on glucose homeostasis as well as other metabolic disturbances in this study (Fig. 11). In addition, the phosphorylation of AMPK was up-regulated by CTRP1 (Fig. 10A, D). As an energy sensor, AMPK could activate GLUT4 and promote the transcription of *G6P*, *PEPCK*, *PGC-1α*, *Gys,* and *GLUT4* [18], which consist of the up-regulation of *G6p* and *Gys2* (Fig. 9C). Moreover, CTRP1 also inhibited the phosphorylation level of ERK (Fig. 10C, F). FIt is reported that activation of the ERK pathway was related to glucose metabolic disorder and systemic insulin resistance, which may result from the transcriptional inhibition of *G6P* and *PEPCK* [20, 24].

The finding that CTRP1 prevented HFD-induced obesity, adipose hypertrophy, and fatty liver is consistent with previous studies. It was previously shown that the body weight of *Ctrp1* transgenic mice was lower than wild-type mice when fed with HFD [13]. In our research, the body weight of CTRP1-treated mice was significantly lower than HFD-fed control mice, as well as the liver and multiple white adipose tissues (Figs. 1–3). CTRP1 also had a beneficial effect on fatty liver and hepatic steatosis (Fig. 3). This is consistent with a previous study that the knockout of *Ctrp1* caused a significant TG accumulation and steatosis in the liver of HFD-fed mice [16]. Moreover, we found that the expression of lipid metabolic genes was changed: De novo fatty acids synthesis gene *Acc1* and lipid uptake gene *Cd36* was decreased; Rate limiting enzyme of triglyceride hydrolysis *Atgl* and rate-limiting enzyme of fatty acid oxidant *Cpt1β*, *Acadm*, *Acadl* was increased (Fig. 9B). These changes at the transcriptional level may result from the activation of AMPK (Fig. 10D). AMPK inhibits SREBP-1c to increase the transcription level of *Acc1* and decreases the phosphorylation of ACC1, thereby inhibiting the downstream signal like *Cpt1β* [18, 25]. Previous studies also indicated that CTRP1 enhanced the phosphorylation of AMPK and ACC in skeletal muscle and activated fat oxidation ex vivo [13, 14].

Energy expenditure is also an important factor in the development of obesity [26]. Consistent with the previous study [13], our results demonstrated that the O_2_ consumption, CO_2_ production, and energy expenditure were elevated in mice of the HFD/CTRP1 group (Fig. 7). Moreover, thermogenic genes also increased in BAT after CTRP1 treatment (Fig. 9D). These suggested that enhanced lipid metabolism and energy consumption contributed to the beneficial effect of CTRP1 in the reduction of adipose hypertrophy and hepatic steatosis. In addition, we observed that overexpression of *CTRP1* reduced the food intake of mice (Fig. 1E, 7L, 8). The paired feeding experiment showed that the body weight gain of HFD-fed control mice was similar to CTRP1-treated mice when the paired amount of food was given (Fig. 8A–C). After withdrawing from the restricted food supply, the body weight gain of control mice elevated and showed significantly faster growth than CTRP1 treated mice (Fig. 8A, B, D). Further, the up-regulation of serum leptin was demonstrated in HFD/CTRP1 group mice (Fig. 8E), suggesting CTRP1 suppressed appetite through leptin. Kubota et al*.* also found that adiponectin could activate AMPK in the arcuate hypothalamus via adipoR1 [27]. However, the transgenic mouse models did not show the appetite-related function of CTRP1 [13, 14, 16]. The circulation level of CTRP1 is higher in our experiment than in transgenic mice [28], which may cause different effects of CTRP1 on the appetite. More study is needed to investigate the mechanism of CTRP1 in appetite regulation and the causal relation between leptin and obesity after CTRP1 treatment.

CTRP1 significantly prevented the development of adipose hypertrophy and hepatic steatosis in HFD-fed mice (Fig. 2D, 3A), but it did not considerably lose weight in obese mice (Fig. 6G). The phosphorylation of AKT was upregulated in EWAT after CTRP1 treatment but not in the liver (Fig. 10B, E). Previous studies also observed controversial evidence between glucose metabolism and lipid clearance in *Ctrp1* KO mice and transgenic mice: *Ctrp1* transgenic mice maintained better glucose homeostasis and lipid homeostasis via AMPK and AKT pathways [13, 14]. Knockout of CTRP1 impaired glucose homeostasis and caused liver steatosis in LFD-fed mice [16]. However, the knockout of CTPR1 improved the lipid metabolism of HFD-fed mice, which may result from the up-regulation of *Scd1*, *Cd36,* and *Pparγ* [16]. We also found that some lipogenesis genes (*Acly*, *Fas*, *Elovl6,* and *Pparγ1*) were up-regulated in our study (Fig. 9A). In addition, the protein level of GLUT4, AMPK, and phosphorylation level of AMPK was decreased in skeletal muscle of LFD-fed *CTRP1* KO mice, but phosphorylation level of AMPK was up-regulated in the liver in HFD-fed *Ctrp1* KO mice [16]. These results suggested that the functions of CTRP1 in regulating lipid metabolism were various in different animal models and pathological statuses.

Hydrodynamic gene delivery of CTRP1 gene generated long-term anti-diabetic effects and beneficial effects in preventing HFD-induced obesity and fatty liver, indicating that CTRP1 has the therapeutic potential in maintaining glucose homeostasis and treating obesity-related metabolic disorders. However, the molecular mechanisms and clear signaling pathways of CTRP1 in preventing obesity, glucose tolerance, and fatty liver remain unclear, as well as the underlying mechanisms of CTRP1 in regulating food intake, inflammation, and thermogenesis. Further studies are needed to investigate the mechanisms of CTRP1 in regulating metabolic homeostasis and appetite. Moreover, the hydrodynamic injection strategy is a physical method of gene delivery to the liver [13, 14, 29]. The overexpressed CTRP1 protein can secrete into the blood and distribute to multiple tissues. CTRP1 was associated with adverse cardiovascular events [30] and was reported to prevent pathological vascular remodeling [31]. CTRP1 also prevented cardiac and renal disease [32, 33]. Further studies are needed to investigate the function of CTRP1 in different animal models, as well as the long-term effects and side effects of CTRP1 in multiple organs, such as cardiovascular systems, bone, muscle, and neurometabolic systems. This will help us better to understand its mechanism at deeper levels.

## Conclusion

This study demonstrates that the hydrodynamic injection of *CTRP1* improves glucose homeostasis and prevents HFD-induced obesity, adipose hypertrophy, and fatty liver due to upregulated energy expenditure, thermogenesis, lipolysis, and reduced inflammation and food intake. The beneficial effects of CTRP1 are related to modulation of the activity of AMPK, AKT, and upregulation of leptin, suggesting CTRP1 may serve as a target in treating metabolic diseases, as well as the potential use of CTRP1 by gene transfer for the treatment of metabolic diseases.

## Supplementary Information

**Additional file 1: Table S1. **Primer list. **Table S2.** Blood index. **Figure S1.** The map of the constructs. **Figure S2.** ALT, AST, and food intake in the HFD fed mice. A. ALT and AST in mice serum at the 11^th^ week of the experiment; B. food intake; C. food intake (kcal). **Figure S3.** Cell viability was detected by CCK8 assay. **Figure S5.** Full view of H&E staining of WAT and BAT. **Figure S5.** Full view of H&E staining and Oil-Red O staining of liver tissue. **Figure S6.** ITT in wight control model on week 8. **Figure S7.** Glucose-stimulated insulin secretion test *in vitro*. **Figure S8.** Body weight, food intake, and fasting blood glucose in STZ-induced T2DM mice. A. The curve of body weight of STZ treated mice; B. Food intake; C. Fasting blood glucose. **Figure S9.** The expression of appetite related genes in the hypothalamus (A) and intestine (B). **Figure S10.** The expression level of upstream regulatory genes of AMPK in liver tissue.

## Data Availability

The datasets used and/or analyzed during the current study are available from the corresponding author on reasonable request.

## Abbreviations

CTRP1: C1q/tumor necrosis factor-related protein 1
STZ: Streptozotocin
HFD: High-fat diet
GTT: Glucose tolerance test
ITT: Insulin tolerance test
HOMA-IR: Homeostatic model assessment for insulin resistance
AST: Aspartate aminotransferase
ALT: Alanine aminotransferase
WAT: White adipose tissue
IWAT: Inguinal white adipose tissue
EWAT: Epididymal white adipose tissue
PWAT: Perirenal white adipose tissue
BAT: Brown adipose tissue
AUC: Area under the curve
T2DM: Type II diabetes
NAFLD: Nonalcoholic fatty liver disease
LFD: Low-fat diet
KO: Knockout
FFA: Free fatty acid
RER: Respiratory exchange ratio
EE: Energy expenditure
