# Supplementary material for: CTRP1 prevents high fat diet-induced obesity and improves glucose homeostasis in obese and STZ-induced diabetic mice
Source: J Transl Med. 2022 Oct 4;20:449. doi: 10.1186/s12967-022-03672-5 (PMC9533627; doi:10.1186/s12967-022-03672-5)
Supplement: Supplementary file 1 — Additional file 1: Table S1. Primer list. Table S2. Blood index. Figure S1. The map of the constructs. Figure S2. ALT, AST, and food intake in the HFD fed mice. A. ALT and AST in mice serum at the 11th week of the experiment; B. food intake; C. food intake (kcal). Figure S3. Cell viability was detected by CCK8 assay. Figure S5. Full view of H&E staining of WAT and BAT. Figure S5. Full view of H&E staining and Oil-Red O staining of liver tissue. Figure S6. ITT in wight control model on week 8. Figure S7. Glucose-stimulated insulin secretion test in vitro. Figure S8. Body weight, food intake, and fasting blood glucose in STZ-induced T2DM mice. A. The curve of body weight of STZ treated mice; B. Food intake; C. Fasting blood glucose. Figure S9. The expression of appetite related genes in the hypothalamus (A) and intestine (B). Figure S10. The expression level of upstream regulatory genes of AMPK in liver tissue. [file 12967_2022_3672_MOESM1_ESM.docx]

**Supplementary Materials and methods**

**CCK8 assay**

CCK8 kit was purchased from Beyotime (Cat. C0037, Shanghai, China). About 5 × 10^3^ HepG2 cells preserved in the laboratory was plated into 96 well plates according to manufacturers’ instructions. Cell was treated with serum of mice hydrodynamic injected with pLIVE-CTRP1 and pLIVE-SEAP (1.125 mg/mL, respectively), or transfected with pLIVE-CTRP1 and pLIVE-SEAP using Lipo2000 (Cat. 11668019, Thermo Fisher Scientific, Waltham, MA, USA) according to manufacturers’ instructions (0.1 mg plasmids per well with 0.1 mL Lipo2000).

**Glucose-stimulated insulin secretion test**

Mice pancreas was injected with 0.5 mg/mL collagenase P in 6 mL Hank’s buffered salt solution (HBSS) with 0.5 mg BSA. Then the pancreas was removed and digested at 37 °C for 12 min. Ten milliliter of HBSS was used for stopping digestion. After washed by 10 mL HBSS for one time, mice islets were isolated under a stereoscopic microscope, and 20 size-matched islets were cultured in RPMI 1640 with 10% fetal bovine serum and 1% penicillin and streptomycin for 16 h.

Cultured size-matched islets were pre-incubated in 500 μL Krebs Ringer Buffer (KRBH, 128 mM NaCl, 10 mM HEPES, 5 mM NaHCO_3_, 4.8 mM KCl, 2.5 mM CaCl_2_, 1.2 mM KH_2_PO_4_, 1.2 mM MgSO_4_ and 0.1% BSA) with 2.8 mM glucose for 30 min. Then the islets were incubated with 500 μL KRBH with 16.7 mM and 2.8 mM glucose plus serum of mice hydrodynamic injected with pLIVE-CTRP1 and pLIVE-SEAP (4.5 mg/mL, respectively) for 30 min. Supernatant insulin was detected by ELISA kits (Cat. ml001983, Mlbio, Shanghai, China).

**Supplementary Table 1 Primer list**

| **Gene** | **Forward primer sequence** | **Reverse primer sequence** | **GeneBank accession number** | **Product length (bp)** | **T_m_** | |
| --- | --- | --- | --- | --- | --- | --- |
|  |  |  |  |  | **F** | **R** |
| *β-actin* | GGCTGTATTCCCCTCCATCG | CCAGTTGGTAACAATGCCATGT | NM_007393 | 154 | 59.96 | 59.44 |
| *Gapdh* | AGGTCGGTGTGAACGGATTTG | TGTAGACCATGTAGTTGAGGTCA | NM_001289726 | 123 | 60.88 | 58.59 |
| *Acc1* | ATGGGCGGAATGGTCTCTTTC | TGGGGACCTTGTCTTCATCAT | NM_133360 | 148 | 60.41 | 58.72 |
| *Acc2* | ACCAGCTTTATCCTGGGCTC | GACGGTGAAATCTCTGTGCAC | NM_001403527 | 246 | 59.45 | 59.54 |
| *Fasn* | GGAGGTGGTGATAGCCGGTAT | TGGGTAATCCATAGAGCCCAG | NM_007988 | 140 | 61.10 | 58.66 |
| *Scd1* | TTCTTGCGATACACTCTGGTGC | CGGGATTGAATGTTCTTGTCGT | NM_009127 | 98 | 60.93 | 59.26 |
| *Fatp2* | CGGTCCGTGACGCAAAT | CCTCCAGCATAGCCAATAA | NM_011978 | 106 | 58.00 | 54.27 |
| *Fabp4* | AAGGTGAAGAGCATCATAACCC | TCACGCCTTTCATAACACATTCC | NM_001409513 | 130 | 58.11 | 59.56 |
| *Lfabp* | CAGAAAGGGAAGGACATCAAG | TGGTCTCCAGTTCGCACTC | NM_017399 | 133 | 56.24 | 59.33 |
| *Elovl3* | TTCTCACGCGGGTTAAAAATGG | GAGCAACAGATAGACGACCAC | NM_001374665 | 220 | 59.77 | 58.47 |
| *Elovl6* | AAGCAGTTCAACGAGAACGAA | CGTACAGCGCAGAAAACAGG | NM_130450 | 79 | 58.45 | 59.84 |
| *Cd36* | ATGGGCTGTGATCGGAACTG | GTCTTCCCAATAAGCATGTCTCC | NM_001159555 | 110 | 60.11 | 59.12 |
| *Mgat1* | TGGTGCCAGTTTGGTTCCAG | TGCTCTGAGGTCGGGTTCA | NM_001110148 | 252 | 60.75 | 60.53 |
| *Pparγ1* | GGAAGACCACTCGCATTCCTT | GTAATCAGCAACCATTGGGTCA | NM_001127330 | 121 | 60.34 | 58.91 |
| *Pparγ2* | TCGCTGATGCACTGCCTATG | GAGAGGTCCACAGAGCTGATT | NM_011146 | 103 | 60.53 | 59.17 |
| *Atgl* | GACAGCTCCACCAACATCCA | GCAAAGGGTTGGGTTGGTTC | NM_001163689 | 196 | 59.96 | 59.89 |
| *Hsl* | TTCGAGGGTGATGAAGGACT | ACTCTGGGTCTATGGCGAAT | NM_001039507 | 238 | 58.06 | 58.21 |
| *Cpt1α* | CTCCGCCTGAGCCATGAAG | CACCAGTGATGATGCCATTCT | NM_013495 | 100 | 60.52 | 58.35 |
| *Cpt1β* | GCACACCAGGCAGTAGCTTT | CAGGAGTTGATTCCAGACAGGT | NM_009948 | 107 | 60.89 | 59.70 |
| *Acadm* | GGGTTTAGTTTTGAGTTGACGG | CCCCGCTTTTGTCATATTCCG | NM_000016 | 109 | 57.78 | 59.67 |
| *Acox* | GAGCAGGAGAAATGGATGCA | GGGCGTAGGTGCCAATTATCT | NM_001271898 | 58 | 57.95 | 60.20 |
| *Acadl* | TCTTTTCCTCGGAGCATGACA | GACCTCTCTACTCACTTCTCCAC | NM_007381 | 113 | 59.38 | 59.31 |
| *Glut2* | TCAGAAGACAAGATCACCGGA | GCTGGTGTGACTGTAAGTGGG | NM_031197 | 215 | 58.47 | 60.88 |
| *Gck* | GTGGTGCTTTTGAGACCCGTT | TTCGATGAAGGTGATTTCGCA | NM_001287386 | 341 | 61.35 | 58.31 |
| *Pklr* | CCGCATCTACATTGACGACG | CCGTGTTCCACTTCGGTCAC | NM_001099779 | 84 | 59.15 | 61.22 |
| *G6p* | CGACTCGCTATCTCCAAGTGA | GTTGAACCAGTCTCCGACCA | NM_008061 | 173 | 59.33 | 59.61 |
| *Pepck* | CTGCATAACGGTCTGGACTTC | CAGCAACTGCCCGTACTCC | NM_011044 | 159 | 58.73 | 60.74 |
| *Acly* | GTGGGGTGATATAGAGTTCCCT | GCTTGCTCCACTTTTGGCATC | NM_001199296 | 94 | 58.41 | 60.67 |
| *Gys2* | CGCTCCTTGTCGGTGACATC | CATCGGCTGTCGTTTTGGC | NM_145572 | 160 | 61.08 | 60.15 |
| *F4/80* | TGACTCACCTTGTGGTCCTAA | CTTCCCAGAATCCAGTCTTTCC | NM_001355722 | 111 | 58.32 | 58.38 |
| *Cd11b* | ATGGACGCTGATGGCAATACC | TCCCCATTCACGTCTCCCA | NM_001082960 | 203 | 60.82 | 60.23 |
| *Cd11c* | CTGGATAGCCTTTCTTCTGCTG | GCACACTGTGTCCGAACTCA | NM_001363984 | 113 | 58.80 | 60.53 |
| *Tnf-α* | CCCTCACACTCAGATCATCTTC | GCTACGACGTGGGCTACAG | NM_013693 | 61 | 57.94 | 60.23 |
| *Il-1β* | GCAACTGTTCCTGAACTCAACT | ATCTTTTGGGGTCCGTCAACT | NM_008361 | 89 | 59.05 | 59.58 |
| *Dio2* | AATTATGCCTCGGAGAAGACCG | GGCAGTTGCCTAGTGAAAGGT | NM_010050 | 125 | 60.22 | 60.55 |
| *Ucp1* | AGGCTTCCAGTACCATTAGGT | CTGAGTGAGGCAAAGCTGATTT | NM_009463 | 133 | 58.15 | 59.18 |
| *Ucp2* | ATGGTTGGTTTCAAGGCCACA | CGGTATCCAGAGGGAAAGTGAT | NM_011671 | 109 | 60.69 | 59.30 |
| *Pgc1α* | TATGGAGTGACATAGAGTGTGC | CCACTTCAATCCACCCAGAAAG | NM_008904 | 134 | 57.34 | 59.18 |
| *Cidea* | TGACATTCATGGGATTGCAGAC | GGCCAGTTGTGATGACTAAGAC | NM_007702 | 171 | 58.98 | 59.00 |
| *Lkb1* | CACTTCACAGTGCCTGGTGTC | AGCAGACAGGGAGCTACACTA | NM_001301853 | 151 | 61.41 | 59.99 |
| *Camkk2* | CTCCCCACAGTCCTCT | AACACCATAGGAGCCCTT | NM_001199676 | 139 | 53.21 | 54.93 |
| *Npy* | GCTCTGCGACACTACATCAA | TGGTTTCAGGGGATGAGATG | NM_023456 | 214 | 58.00 | 56.89 |
| *Pomc* | AGCAACCCGCCCAAGG | GCGTCTGGCTCTTCTCGG | NM_001278581 | 61 | 59.52 | 60.20 |
| *Agrp* | AGGGCATCAGAAGGCCTGACCAGG | CATTGAAGAAGCGGCAGTAGCACGT | NM_001271806 | 255 | 68.20 | 65.95 |
| *Cck* | CGCTGGAACTCGCCAAGCCA | GCGGCCAGAAGGAGCTTTGC | NM_031161 | 270 | 65.77 | 64.63 |
| *Glp-1* | TGAAGACAAACGCCAC | TCATGACGTTTGGCAA | NM_008100 | 135 | 51.63 | 50.67 |
| *Pyy* | GGACGCCTACCCTGCCAAACCA | AGTGCCCTCTTCTTAAACCAAACA | NM_145435 | 298 | 67.03 | 60.63 |

**Supplementary Table 2 blood index**

|  | **ALT** | **AST** | **TBIL** | **TP** | **ALB** | **GLB** | **A/G** | **CREA** | **Urea** | **UA** | **LDH** |
| --- | --- | --- | --- | --- | --- | --- | --- | --- | --- | --- | --- |
| HFD/SEAP | 41.08±12.66 | 186.4±36.70 | 5.133±0.53 | 63.8±4.74 | 30.44±4.04 | 36.2 | 0.77±0.014 | 13.3±3.82 | 8.5±0.94 | 94.2±27.09 | 672.8±167.26 |
| HFD/CTRP1 | 43.8±4.20 | 172.6±81.56 | 5.067±0.61 | 66.68±7.69 | 32.08±0.33 | 38.2±8.20 | 0.77±0.014 | 15±6.08 | 9.08±0.48 | 89.52±35.13 | 676.8±307.58 |
| p-value | 0.704767 | 0.738434 | 0.880761 | 0.491268 | 0.416525 | 0.610947 | >0.999999 | 0.605542 | 0.223952 | 0.840621 | 0.982196 |


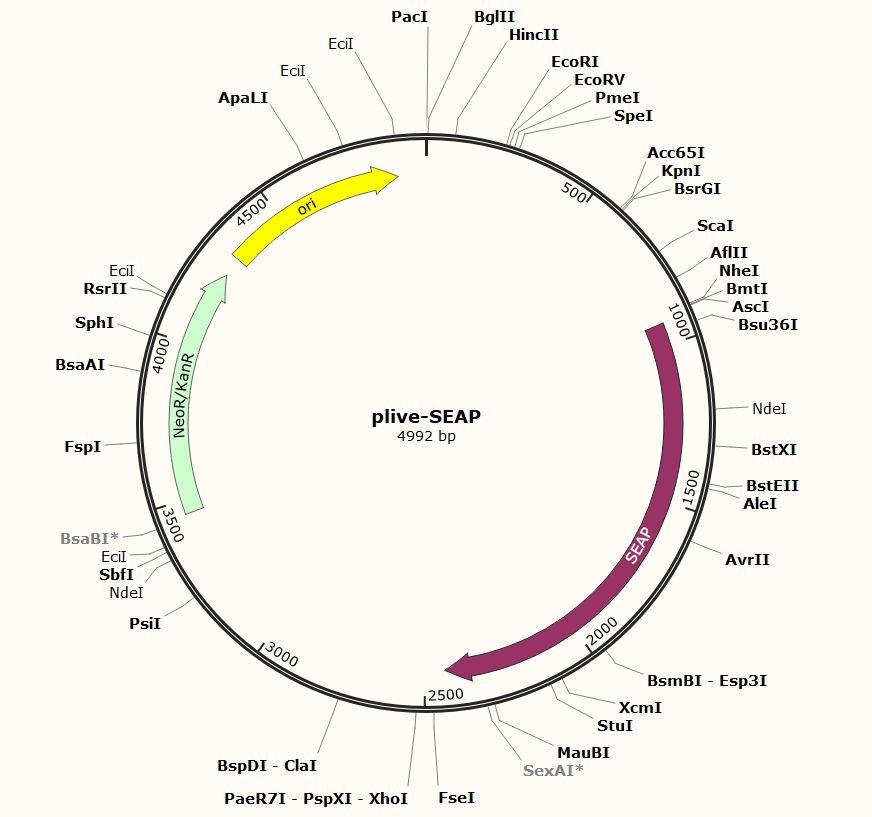

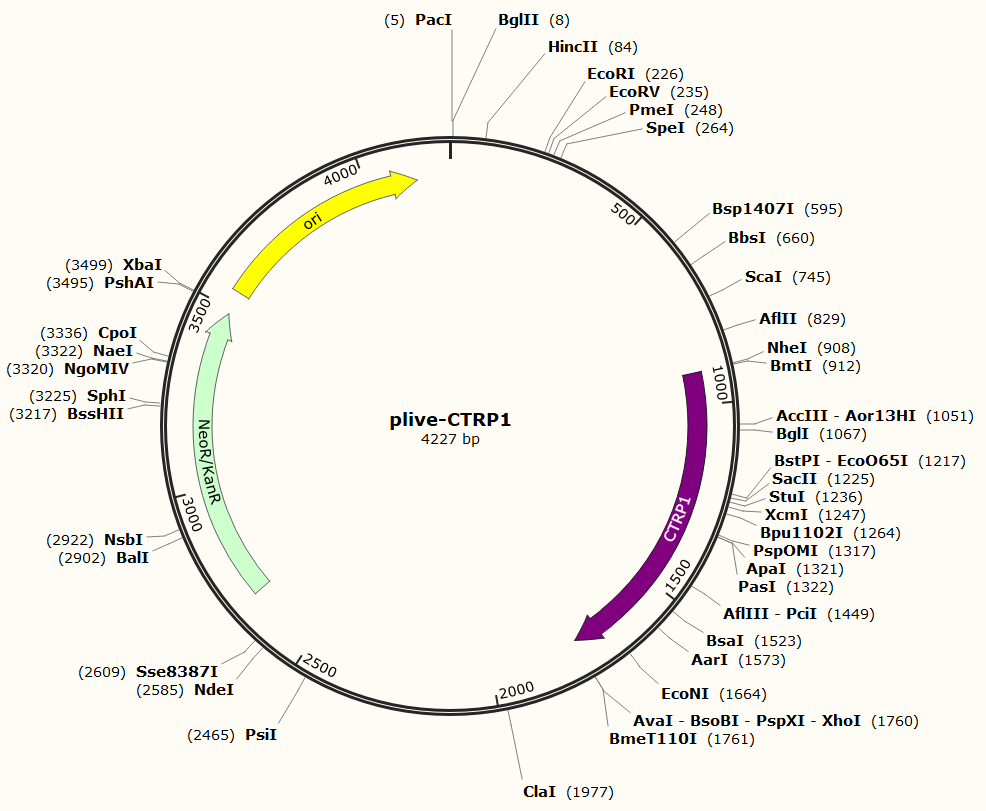


**Supplementary Fig. 1. The map of the constructs.**


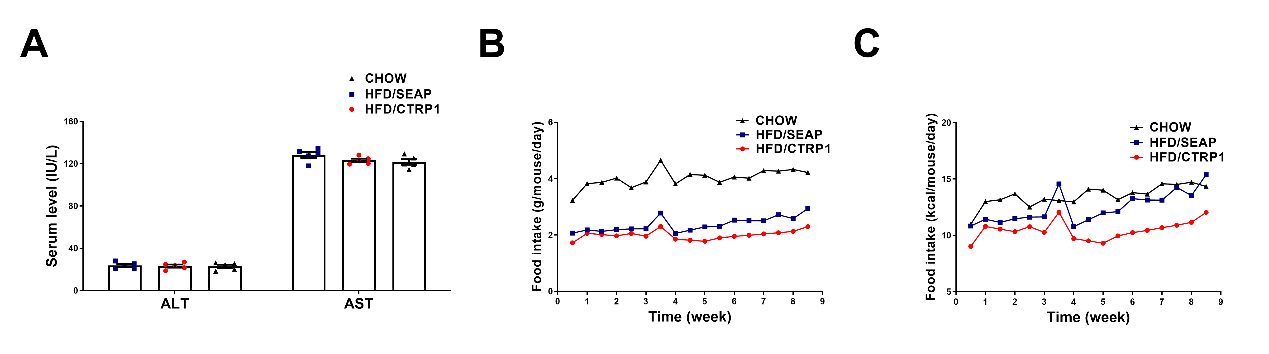


**Supplementary Fig. 2. ALT, AST, and food intake in the HFD fed mice.** A. ALT and AST in mice serum at the 11^th^ week of the experiment; B. food intake; C. food intake (kcal).


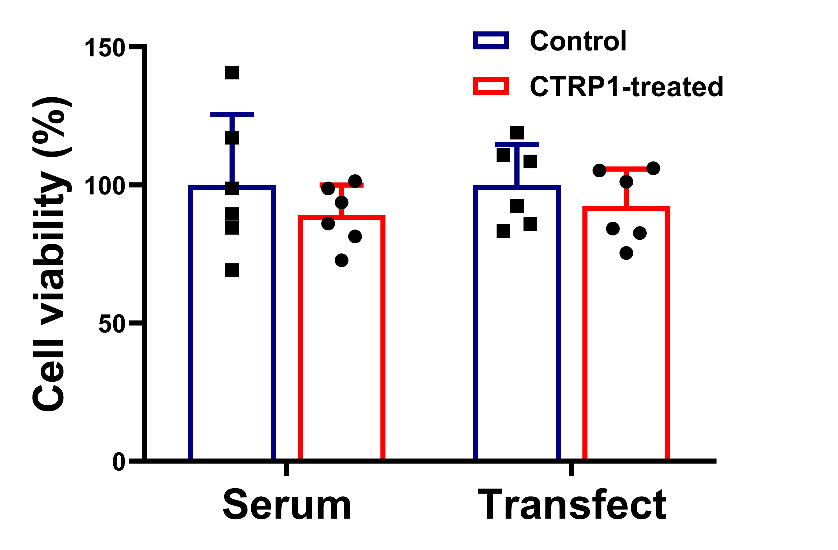


**Supplementary Fig. 3. Cell viability was detected by CCK8 assay.**

**Supplementary Fig. 5. Full view of H&E staining of WAT and BAT.**


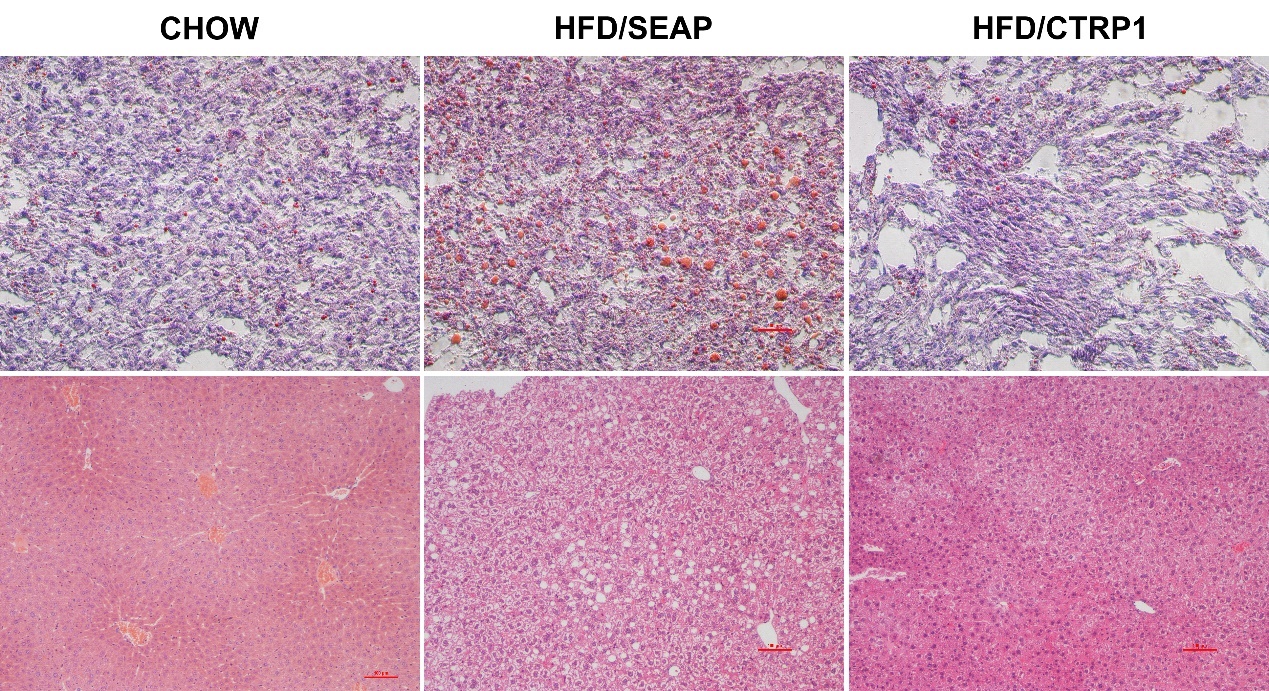


**Supplementary Fig. 5. Full view of H&E staining and Oil-Red O staining of liver tissue.**


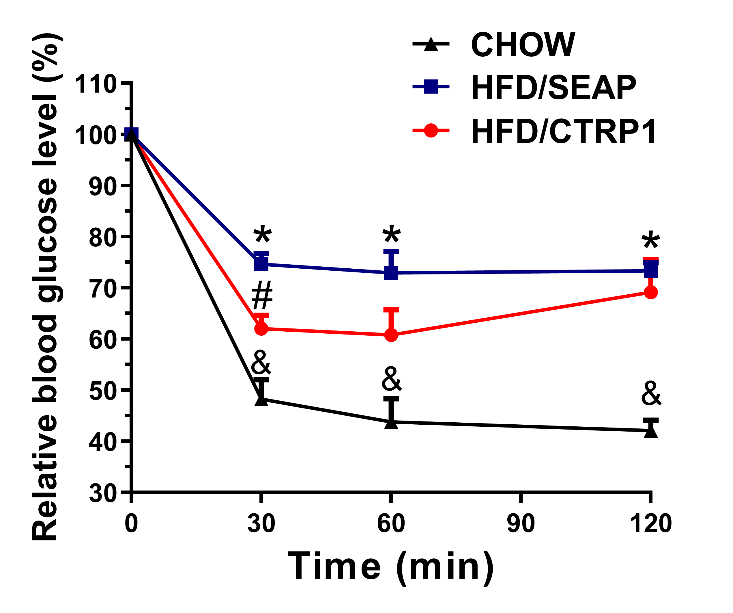


**Supplementary Fig. 6. ITT in wight control model on week 8.**


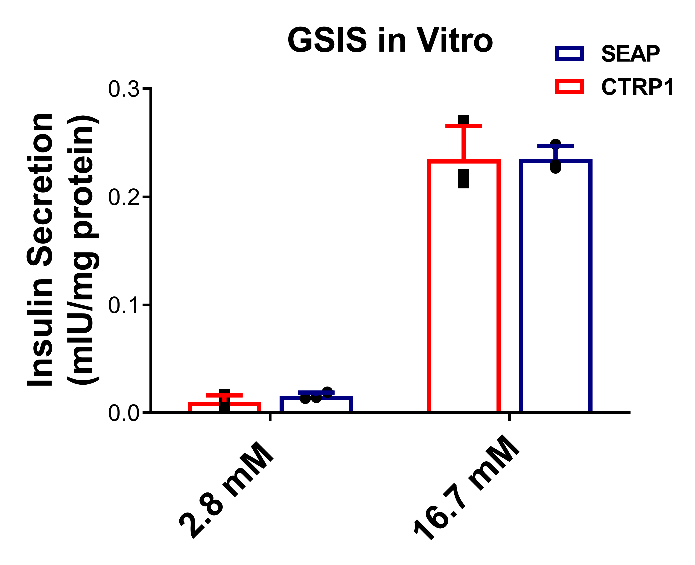


**Supplementary Fig. 7. Glucose-stimulated insulin secretion test *in vitro*.**


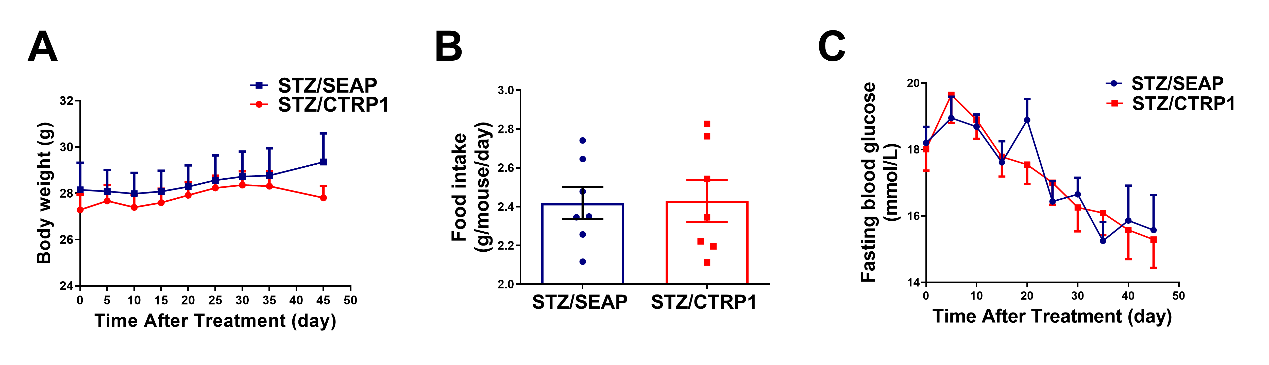


**Supplementary Fig. 8. Body weight, food intake, and fasting blood glucose in STZ-induced T2DM mice.** A. The curve of body weight of STZ treated mice; B. Food intake; C. Fasting blood glucose.


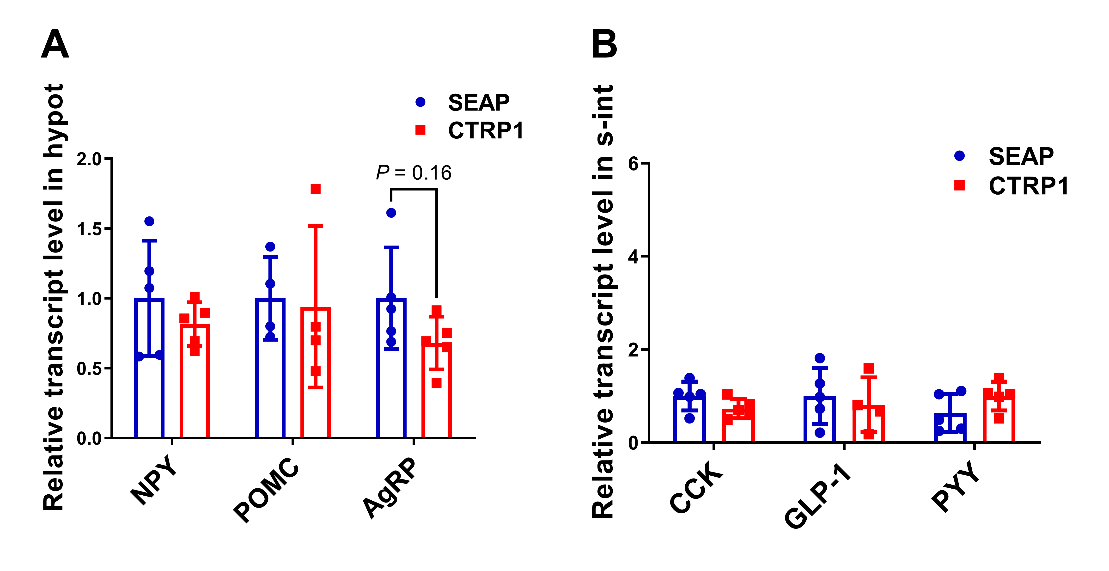


**Supplementary Fig. 9. The expression of appetite related genes in the hypothalamus (A) and intestine (B).**


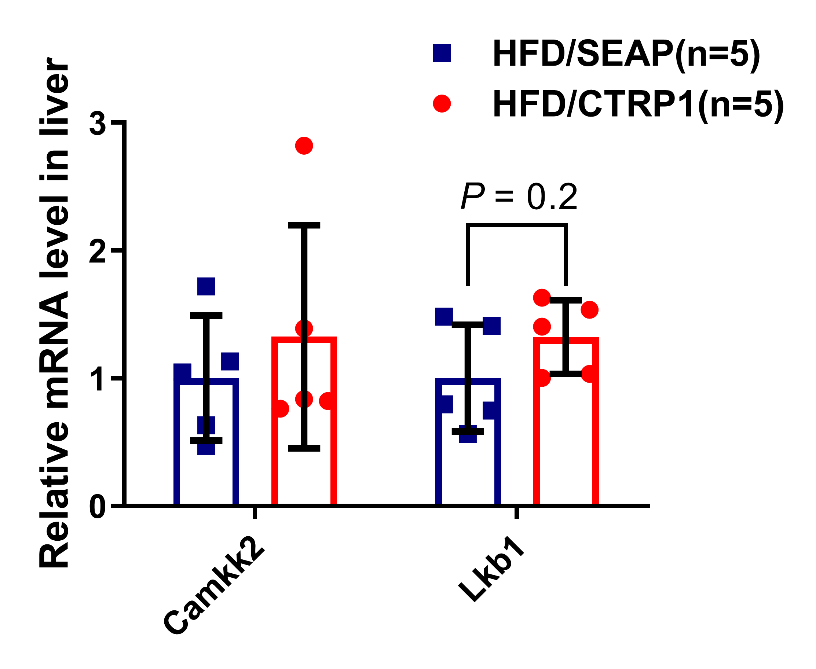


**Supplementary Fig. 10. The expression level of upstream regulatory genes of AMPK in liver tissue.**
